# Structural determinants of dual incretin receptor agonism by tirzepatide

**DOI:** 10.1073/pnas.2116506119

**Published:** 2022-03-25

**Authors:** Bingfa Sun, Francis S. Willard, Dan Feng, Jorge Alsina-Fernandez, Qi Chen, Michal Vieth, Joseph D. Ho, Aaron D. Showalter, Cynthia Stutsman, Liyun Ding, Todd M. Suter, James D. Dunbar, John W. Carpenter, Faiz Ahmad Mohammed, Eitaro Aihara, Robert A. Brown, Ana B. Bueno, Paul J. Emmerson, Julie S. Moyers, Tong Sun Kobilka, Matthew P. Coghlan, Brian K. Kobilka, Kyle W. Sloop

**Affiliations:** ^a^ConfometRx, Santa Clara, CA 95054;; ^b^Molecular Pharmacology, Lilly Research Laboratories, Eli Lilly and Company, Indianapolis, IN 46285;; ^c^BioTechnology Discovery Research, Lilly Research Laboratories, Eli Lilly and Company, Indianapolis, IN 46285;; ^d^Discovery Chemistry Research and Technologies, Lilly Research Laboratories, Eli Lilly and Company, Indianapolis, IN 46285;; ^e^Lilly Biotechnology Center San Diego, San Diego, CA 92121;; ^f^Diabetes and Complications, Lilly Research Laboratories, Eli Lilly and Company, Indianapolis, IN 46285;; ^g^Discovery Chemistry Research and Technologies, Lilly, S.A., 28108 Alcobendas, Madrid, Spain

**Keywords:** G protein coupled receptor (GPCR), structure, tirzepatide, GIP receptor, GLP-1 receptor

## Abstract

Tirzepatide (LY3298176) is a fatty-acid-modified, dual incretin receptor agonist that exhibits pharmacology similar to native GIP at the glucose-dependent insulinotropic polypeptide receptor (GIPR) but shows bias toward cyclic adenosine monophosphate signaling at the glucagon-like peptide-1 receptor (GLP-1R). In addition to GIPR signaling, the pathway bias at the GLP-1R may contribute to the efficacy of tirzepatide at improving glucose control and body weight regulation in type 2 diabetes mellitus. To investigate the structural basis for the differential signaling of tirzepatide, mechanistic pharmacology studies were allied with cryogenic electron microscopy. Here, we report high-resolution structures of tirzepatide in complex with the GIPR and GLP-1R. Similar to the native ligands, tirzepatide adopts an α-helical conformation with the N terminus reaching deep within the transmembrane core of both receptors. Analyses of the N-terminal tyrosine (Tyr1^Tzp^) of tirzepatide revealed a weak interaction with the GLP-1R. Molecular dynamics simulations indicated a greater propensity of intermittent hydrogen bonding between the lipid moiety of tirzepatide and the GIPR versus the GLP-1R, consistent with a more compact tirzepatide–GIPR complex. Informed by these analyses, tirzepatide was deconstructed, revealing a peptide structure–activity relationship that is influenced by acylation-dependent signal transduction. For the GIPR, Tyr1^Tzp^ and other residues making strong interactions within the receptor core allow tirzepatide to tolerate fatty acid modification, yielding an affinity equaling that of GIP. Conversely, high-affinity binding with the extracellular domain of the GLP-1R, coupled with decreased stability from the Tyr1^Tzp^ and the lipid moiety, foster biased signaling and reduced receptor desensitization. Together, these studies inform the structural determinants underlying the function of tirzepatide.

## Significance

Tirzepatide is a dual agonist of the glucose-dependent insulinotropic polypeptide receptor (GIPR) and the glucagon-like peptide-1 receptor (GLP-1R), which are incretin receptors that regulate carbohydrate metabolism. This investigational agent has proven superior to selective GLP-1R agonists in clinical trials in subjects with type 2 diabetes mellitus. Intriguingly, although tirzepatide closely resembles native GIP in how it activates the GIPR, it differs markedly from GLP-1 in its activation of the GLP-1R, resulting in less agonist-induced receptor desensitization. We report how cryogenic electron microscopy and molecular dynamics simulations inform the structural basis for the unique pharmacology of tirzepatide. These studies reveal the extent to which fatty acid modification, combined with amino acid sequence, determines the mode of action of a multireceptor agonist.

Designing therapeutic ligands capable of targeting multiple receptor systems offers opportunities to discover more effective treatments for complex diseases, especially for conditions where intervening at more than one signaling pathway may be beneficial. One such molecule is tirzepatide (LY3298176), a 39-amino acid linear peptide possessing agonist activity at both the glucose-dependent insulinotropic polypeptide receptor (GIPR) and the glucagon-like peptide-1 receptor (GLP-1R) (1, 2). The dual agonist nature of this molecule represents a promising therapeutic modality for the treatment of metabolic disorders, such as type 2 diabetes mellitus (T2DM), obesity (including heart failure patients with preserved ejection fraction), and nonalcoholic steatohepatitis. This therapeutic approach is founded on the established clinical efficacy of selective GLP-1R agonists, with the bifunctional concept being informed by the hypothesis that concerted activation of both receptors improves glucose control, energy balance, and lipid storage (3, 4). In addition to the dual pharmacology, maintaining efficacious concentrations of the drug is important for maximizing the benefit of this type of treatment. Therefore, to sustain the actions of tirzepatide, the peptide is conjugated through a lysine located near the middle of the molecule to a C20 fatty diacid moiety via a hydrophilic linker (2). This fatty acid modification enables reversible, noncovalent binding to human serum albumin and thus contributes to a pharmacokinetic profile that enables once-weekly dosing of the drug (1).

To date, the clinical development program for tirzepatide has yielded encouraging results, highlighted by data showing improvements in glycemic control and energy metabolism. The efficacy of tirzepatide to effectively lower glucose and body weight in subjects with T2DM was established in a 26-wk phase 2b trial (5). Moreover, post hoc analyses of this trial reported that treatment with tirzepatide demonstrated favorable effects on markers of insulin sensitivity and pancreatic beta cell function and also reduced atherogenic lipid particles (6, 7). Interestingly, the improvement in insulin sensitivity appeared largely independent of changes in body weight (6). These data supported the initiation of a phase 3 clinical development program for tirzepatide, known as SURPASS (8), and in particular, the results reported from SURPASS-2, a 40-wk pivotal trial in subjects with T2DM, indicate the benefit of dual GIP/GLP-1 pharmacology (9). In this head-to-head study against the highest dose of the GLP-1R monoagonist semaglutide that is currently approved, all three doses of tirzepatide delivered superior glucose and weight reductions. The superior clinical efficacy of tirzepatide points to the advantage of adding GIP pharmacology to GLP-1 therapy.

Due to the clinical data, determining the mechanisms responsible for the improvement in metabolic control that occur upon treatment with tirzepatide is an area of active investigation. Ex vivo assays using islets isolated from *Gipr* or *Glp-1r* knockout mice and glucose tolerance tests performed in both of these models demonstrate that tirzepatide can enhance insulin secretion and reduce hyperglycemia through either receptor (1). Pharmacologically, receptor-specific cyclic adenosine monophosphate (cAMP) accumulation assays show tirzepatide is an imbalanced agonist favoring GIPR over GLP-1R activity; these results align with binding data indicating the affinity of tirzepatide for the GIPR is equal to that of GIP but approximately fivefold weaker than GLP-1 on the GLP-1R (1, 10). The benefit of strong GIPR activity is supported by findings from studies showing that GIPR agonism improves insulin sensitivity by a mechanism independent of weight loss (11). Furthermore, an intact GIP axis in the brain is necessary to achieve the full anorexigenic effect of dual GIPR and GLP-1R agonist treatment (12). In line with both of these findings, the expression of the GIPR in adipose tissue and certain metabolic control centers in the brain likely contribute to the benefit of the GIP component of tirzepatide (3).

At the same time, studies characterizing the pharmacology of tirzepatide at the GLP-1R have garnered attention by showing that it displays pathway bias for cAMP signaling over β-arrestin recruitment (10, 13). The significance of this finding is not fully realized but may be substantive in light of reports showing biased analogs of the GLP-1R agonist exenatide are more efficacious than the nonbiased parent molecule in rodent models (14, 15). Together, the prevailing evidence supports a therapeutic benefit of combining potent GIPR agonism with biased GLP-1R signaling, aligning with the promising outcomes of the clinical studies for tirzepatide. Thus, the experiments performed and presented herein were undertaken to better understand the unique ligand-binding and pharmacological characteristics of tirzepatide on both the GIPR and the GLP-1R at the molecular level.

## Results and Discussion

### Cryogenic Electron Microscopy (Cryo-EM) Structures of Tirzepatide in Complex with the GIPR and GLP-1R.

**Fig. 1.**
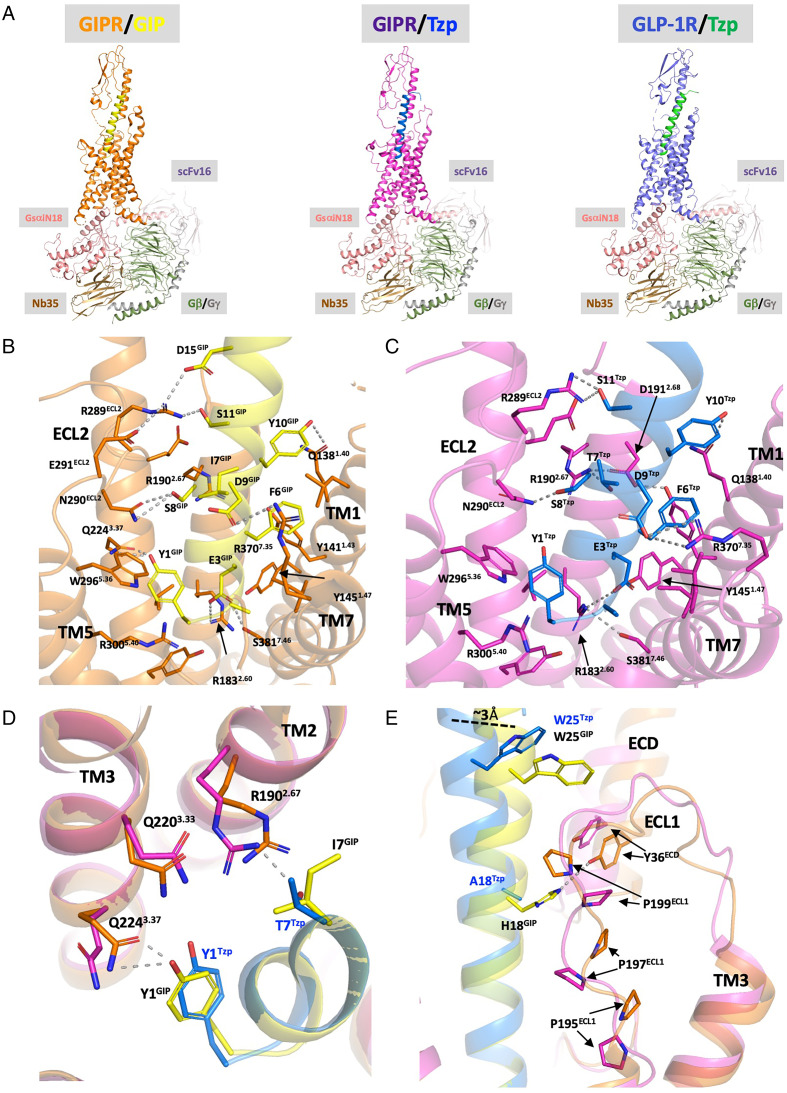
Cryo-EM structure determination of the GIPR/GIP, GIPR/tirzepatide, and GLP-1R/tirzepatide. (*A*) Overall structures of GIPR (orange)/GIP (yellow) (3.2-Å resolution), GIPR (magenta)/tirzepatide (blue) (3.1-Å resolution), and GLP-1R (slate blue)/tirzepatide (green) (2.9-Å resolution). Additional subunits of the complexes are colored as the following: GsαiN18, salmon; Gβ, dark green; Gγ, gray; Nb35, brown; ScFv16, violet-brown. (*B*, *C*) The interaction of GIP (*B*) or tirzepatide (*C*) and the TM domain of GIPR. Residues that are involved in interactions are shown as sticks, and the residues that contribute most significant interactions are labeled. Hydrogen bonds were labeled as dashes. (*D*) Difference of the residue on position 7 of GIP and tirzepatide. (*E*) The interaction of GIP and tirzepatide with the ECD and ECL1 of the GIPR.

**Fig. 2.**
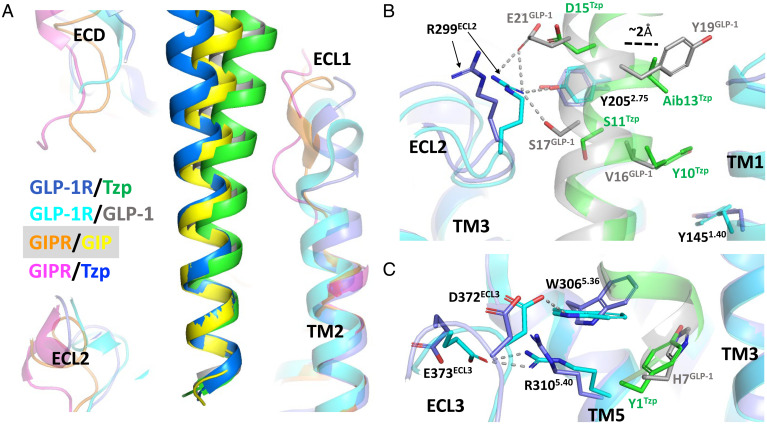
Differential binding of tirzepatide (TZP) versus GLP-1 at the GLP-1R. (*A*) The overall orientation of TZP bound to the GLP-1R (GLP-1R slate blue; Tzp, green) compared with GLP-1R (cyan)/GLP-1 (gray) (PDB: 6 × 18), GIPR (orange)/GIP (yellow), and GIPR (magenta)/Tzp (blue). (*B*) TZP is positioned further away from the ECL2 of the GLP-1R, and Arg299^ECL2^ does not interact with TZP. Residues that are involved in significant interactions are shown as sticks. Hydrogen bonds are labeled as dashes. (*C*) The bulkiness of Tyr1^Tzp^ results in a different rotamer of Trp306^5.36^, disrupting the interactions between TM5 and ECL3 of the GLP-1R.

To investigate the molecular basis for the pharmacological characteristics of tirzepatide, high-resolution structures of the GIPR in complex with native GIP (3.2-Å resolution) and of the GIPR and GLP-1R bound to tirzepatide (3.1-Å and 2.9-Å resolution, respectively) were determined by cryo-EM (Fig. 1*A* and *SI Appendix*, Figs. S1 and S2 and Table S1). The ligands were confidently modeled to the density maps (*SI Appendix*, Figs. S1 and S2), and residues 1 to 32 of both GIP and tirzepatide were resolved in the structures, comparable with the length of GLP-1 resolved in previously reported structures of the GLP-1R (16, 17). Importantly, the GIP/GIPR structure establishes the activation mechanism for this receptor, and similar to GLP-1 and glucagon in complex with their respective receptors, GIP adopts a continuous helical conformation (16, 18). The N terminus inserts into the transmembrane (TM) domain, making contacts with residues in TM1, TM2, TM3, TM5, TM7, and extracellular loop 2 (ECL2) through extensive polar and hydrophobic interactions (Fig. 1 *A* and *B*). Of note, tyrosine in position 1 (Tyr1^GIP^) is buried in the TM core of the GIPR, hydrogen bonding with Gln224^3.37^ and making aromatic interactions with Trp296^5.36^ (Fig. 1*B*). The critical role of Tyr1 in GIP binding and receptor activation is supported by studies of truncated or mutant analogs of GIP (19, 20). Analogous results and conclusions with respect to the determinants of the GIP/GIPR interaction have recently been described (21). Consistent with its sequence similarity with the N-terminal portion of GIP, tirzepatide binds the GIPR in an analogous manner (Fig. 1 *A* and *C*). The main chain of the N-terminal segment (∼residues 1 to 14) of tirzepatide largely overlaps with the equivalent segment of GIP (Fig. 1 *D* and *E*). Although most interactions of tirzepatide and GIP are similar, a key difference is the threonine at position 7 (Thr7^Tzp^) of tirzepatide (versus isoleucine in GIP) that provides hydrogen bonding with Arg190^2.67GIPR^ (Fig. 1*D*), mimicking the interaction between the equivalent Thr13^GLP-1^ and Lys197^2.67GLP-1R^ (17). Furthermore, the side chain of Arg190^2.67GIPR^ is in a slightly different conformation compared to the GIP-bound structure, bringing it closer to Tyr1^Tzp^. With its side chain adopting a different rotamer, the hydroxyl group of Tyr1^Tzp^ points toward Arg190^2.67GIPR^ and Gln220^3.33GIPR^ (Fig. 1*D*), and the cryo-EM map suggests there are water-mediated polar interactions among these residues (*SI Appendix*, Fig. S3). The extra interactions indicate that the sequence of tirzepatide may foster higher affinity and potency properties at the GIPR. Fig. 2.

The C terminus of GIP (∼residues 15 to 32) interacts with the extracellular domain (ECD) and ECL1 and, overall, resembles the interaction pattern of GLP-1 bound to the GLP-1R (16). The ECD engages GIP in the same way as prior crystallography studies of the isolated ECD of the GIPR indicated (22), and our studies provide a comprehensive understanding by showing that the ECL1 of the GIPR also contributes to the interaction with the C-terminal region of GIP, most notably His18^GIP^ and Trp25^GIP^ (Fig. 1*E*). ECL1 adopts a more extended conformation in GIPR compared with that for both the GLP-1R (16) and the glucagon receptor (GCGR) (23), where an alpha helical structure is formed. The presence of Pro195^GIPR^, Pro197 ^GIPR^, Pro199^GIPR^ in the ECL1 prevents formation of a stable secondary structure (Fig. 1*E*). A conformation of ECL1 similar to that in the GIP-bound structure is observed for tirzepatide (Fig. 1*E*), but the C-terminal segment of tirzepatide tilts away from ECL1 for about 3 Å at the Cα of Trp25 compared with the equivalent Cα of GIP (Fig. 1*E*). In addition, a hydrogen bond is formed by His18^GIP^ of GIP and Tyr36^ECD^ of the GIPR, which does not occur with tirzepatide because it contains Ala18^Tzp^ (Fig. 1*E*). Interestingly, the ECD-TM1 linker is better resolved in the GIPR/tirzepatide structure versus that with GIP, suggesting less flexibility (*SI Appendix*, Fig. S4). Also of note is that the side chain of Lys20^Tzp^ extends toward Leu128^1.30GIPR^ at the end of the ECD-TM1 linker (*SI Appendix*, Fig. S4). As anticipated, the fatty acid moiety attached to Lys20^Tzp^ is not resolved in the density map.

Overall, the helices of GIP and tirzepatide are positioned further away from ECL2 and closer to TM1 in the GIPR compared with the location of GLP-1 bound to the GLP-1R, but most other components of the TM for the GIPR and GLP-1R are well aligned (*SI Appendix*, Fig. S5). At this position, a hydrogen bond forms between Tyr10^GIP^ or Tyr10^Tzp^ and the Gln138^1.40GIPR^ (Fig. 1 *B* and *C*). By contrast, the equivalent Val16^GLP-1^ does not interact with Tyr145^1.40GLP-1R^ (17).

Although the GIPR/tirzepatide structure largely resembles the GIPR/GIP structure, the GLP-1R/tirzepatide structure reveals that tirzepatide engages the GLP-1R in a distinct manner (Fig. 2). Alignment of the structures by the TMs shows the N termini of the ligands occupy the same space, but the C-terminal segment of tirzepatide in complex with the GLP-1R tilts toward its ECL1 (Fig. 2*A* and *SI Appendix*, Fig. S6*A*), which adopts a conformation similar to that found in the GLP-1R/Ex-P5 structure (24) (*SI Appendix*, Fig. S6*B*) and the GLP-1R/taspoglutide complex (25). The flexibility of the GLP-1R-ECL1 conformation accommodates ligands with different sequences, and in all of these, Trp214^ECL1^ makes an important pi–pi interaction with the aromatic residues of the Phe-X-X-Trp motif of the peptides (*SI Appendix*, Fig. S6*C*).

**Fig. 3.**
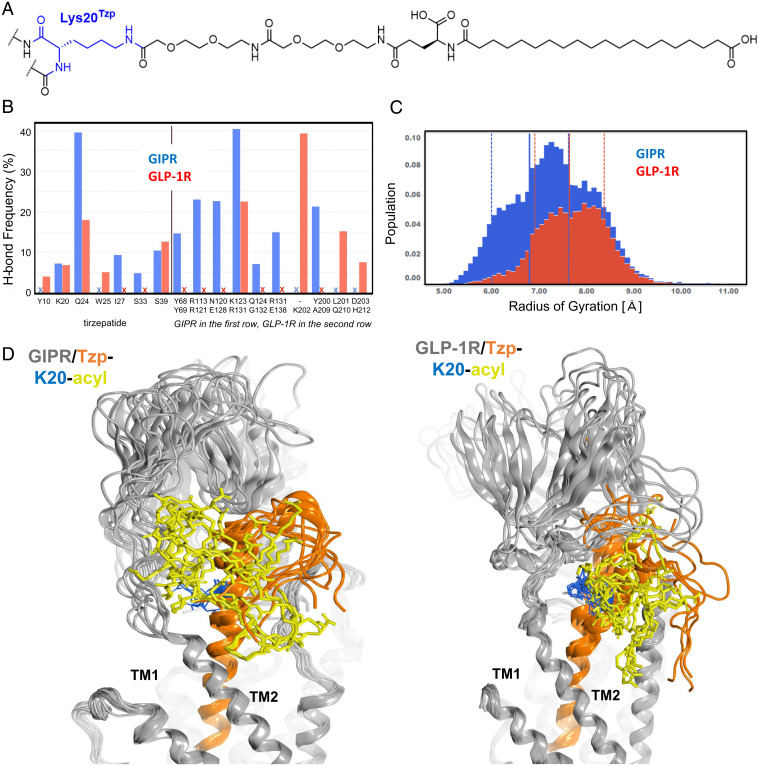
MD simulations of the GIPR and the GLP-1R in complex with TZP. (*A*) Chemical structure of the lipid modification and its attachment to Lys20^Tzp^ of TZP. (*B*) Frequency of hydrogen bond interactions observed during the MD simulations by the lipid chain with the peptide sequence of TZP (*Left* panel) or the receptors (*Right* panel). Results for the GIPR are represented by the blue bars and the GLP-1R data as red. “X” denotes the absence of hydrogen bond interactions of the corresponding residues in the receptor complex. (*C*) The radius of gyration distribution of the lipid chain over two 500-nsec MD runs in the GIPR (blue) and GLP-1R (red) complexes, respectively. (*D*) Overlays of MD snapshots of TZP in GIPR (*Left*) and GLP-1R (*Right*). Ten snapshots at 100-nsec intervals from each complex system are displayed. The peptide portion of TZP is shown as orange ribbons, and the lipid chain is shown as yellow sticks.

Despite some general similarity of the overall orientation of tirzepatide and GLP-1, detailed analyses of the GLP-1R/tirzepatide and GLP-1R/GLP-1 structures reveal differences in their binding, consistent with the weaker affinity of tirzepatide (10, 16). The Cα atoms of residues 2 to 9 of tirzepatide align well with the equivalent amino acids of GLP-1 (residues 8 to 15), all within 1 Å of each other. From Tyr10^Tzp^ onward, the distance between their equivalent Cα atoms increases to about 2.2 Å within the subsequent helical turn at 2-aminoisobutyric acid (Aib)13^Tzp^ and Tyr19^GLP-1^ (Fig. 2*B*). Although this is a modest difference, the shift in the position of tirzepatide results in weaker interactions with the ECL2 of the GLP-1R, which is implicated in determining the signaling profile of GLP-1R (26, 27). Specifically, Arg299^ECL2^, which mediates polar interactions with various GLP-1R ligands (17), does not form such contacts with tirzepatide (Fig. 2*B* and *SI Appendix*, Fig. S7*A*). Mutation of Arg299^ECL2^ to alanine reduces the efficacy of GLP-1 for activating cAMP signaling (26, 27). On the other hand, the relative shift in the position of tirzepatide allows pi–pi stacking between Tyr10^Tzp^ and Tyr145^1.40GLP-1R^, mimicking the interaction mode of oxyntomodulin by its equivalent Tyr10 residue (28) (Fig. 2*B*).

The N terminus of tirzepatide presents another key feature which may contribute to its weaker affinity for binding the GLP-1R. Due to the bulkier side chain of Tyr1^Tzp^ (versus His7^GLP-1^), Trp306^5.36GLP-1R^ adopts an alternate rotamer which ensues steric conflict relative to the conformation of the GLP-1R/GLP-1 structure (Fig. 2*C* and *SI Appendix*, Fig. S7*B*). This change disrupts hydrogen bonding between Trp306^5.36^ and Asp372^ECL3^ that is observed in the GLP-1R/GLP-1 structure (Fig. 2*C*), similar to the scenario in the GIPR/GIP and GIPR/tirzepatide structures (*SI Appendix*, Fig. S7*C*). This specific conformation of Trp306^5.36GLP-1R^ also changes conformations of many of the surrounding residues, including residues of the GLP-1R-ECL2 and Arg310^5.40GLP-1R^. Furthermore, the polar interaction between Arg310^5.40^ and Glu373^ECL3^ in the GLP-1R/GLP-1 structure is also lost in the tirzepatide bound structure (Fig. 2*C*). Together, polar interactions between the TM5 and ECL3 of the GLP-1R are lost due to the presence of tyrosine versus histidine at position 1. A previous report suggested that stabilization of ECL3 is required for full, unbiased agonism of the GLP-1R (29). Consistent with the loss of stabilizing interactions, the ECL3 in the GLP-1R/tirzepatide structure exhibits weaker density in the cryo-EM map. Notably, although fully buried in the pocket, the density for Tyr1^Tzp^ and Trp306^5.36GLP-1R^ is weaker than other residues of tirzepatide for its binding pocket (*SI Appendix*, Fig. S7*D*), reflecting their flexibility in this less stable conformation.

### Dynamics of the Linker–Fatty Acid Moiety of Tirzepatide.

A key structural feature of tirzepatide is the C20 fatty diacid attached to the side chain nitrogen of Lys20^Tzp^ via a linker of a L-γ-glutamic acid and two 8-amino-3,6-dioxaoctanoic acids (2) (Fig. 3*A*). In the tirzepatide-complexed GLP-1R and GIPR cryo-EM structures, both the linker–fatty acid moiety and the peptide C terminus (amino acids 33 to 39, hereinafter referred to as the Ct) appear disordered and were not resolved. Although structural aspects of acylated peptide interaction with the GLP-1R have not been previously resolved by crystallography (30), the recent cryo-EM structure of semaglutide (which contains a C18 diacid at position 20) bound to the GLP-1R revealed some density for the lipid modification (25). In this report on semaglutide, two conformations of linker–fatty acid chain were modeled, namely, one interacting with the ECD and one with the membrane (25).

**Fig. 4.**
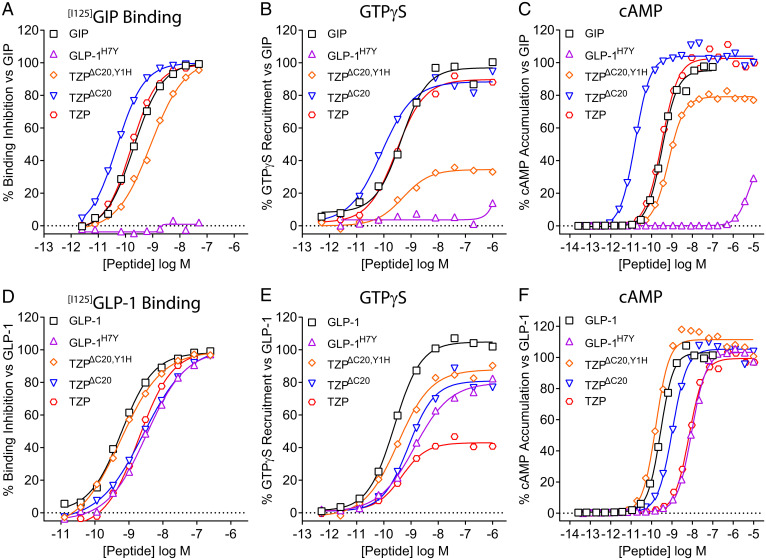
The C20 diacid fatty acid moiety of TZP impacts incretin receptor binding affinity. Mechanistic pharmacology studies investigating receptor binding and signal transduction were performed using TZP, an analog thereof lacking the lipid moiety (TZP^ΔC20^), and a derivative that also contains histidine in place of tyrosine at position 1 (TZP^ΔC20,Y1H^). (*A*) For each ligand, competitive inhibition of [^125^I]-GIP (1–42) binding was determined using membranes isolated from HEK293 cells expressing the human GIPR. Binding of TZP is shown to be equivalent to that of native GIP. Removal of the C20 diacid fatty acid chain increased the affinity of the ligand, while changing tyrosine to histidine weakened binding to the receptor. (*B*) Ligand-induced GTPγS binding of Gα_s_ was performed using the GIPR-expressing membranes. Removal of the lipid moiety from TZP resulted in a modest increase in potency for inducing activation of the Gα_s_ versus TZP and GIP, but the TZP^ΔC20,Y1H^ analog showed both reduced efficacy and potency in comparison. (*C*) Agonist-stimulated cAMP production was measured in human GIPR expressing HEK293 cells. In line with the increase in binding affinity and potency to activate Gα_s_, absence of the lipid moiety led to increased potency for stimulating GIPR-mediated cAMP accumulation, while weaker activity was observed upon replacement of the tyrosine at position 1. (*D*) For the GLP-1R, competitive inhibition of [^125^I]-GLP-1(7-36)NH_2_ binding was determined using membranes isolated from HEK293 cells expressing the human GLP-1R. Binding of both TZP and TZP^ΔC20^ is shown to be ∼fivefold weaker than that of GLP-1, but changing tyrosine to histidine at position 1 restored the binding affinity to that of the native peptide. (*E*) Ligand-induced GTPγS binding of Gα_s_ was performed using the GLP-1R-expressing membranes. Compared with GLP-1, TZP is shown to be a partial agonist at stimulating Gα_s_. Removal of the lipid moiety from TZP resulted in an increase in the efficacious response, with a slightly further elevation observed for the TZP^ΔC20,Y1H^ analog. (*F*) Agonist-stimulated cAMP production was measured in human GLP-1R-expressing HEK293 cells. The potency of TZP at stimulating cAMP accumulation is ∼20-fold weaker than that of GLP-1. The absence of the lipid moiety improved potency and the nonlipidated parent peptide containing the histidine displayed activity that is indistinguishable from that of GLP-1. A derivative of glucagon-like peptide-1 containing tyrosine in place of histidine at position 7 (GLP-1^H7Y^) was used as a control. Data presented are representative of *n* ≥ 3 independent experiments. Summarized data are shown in *SI Appendix*, Table 2. log M; log Molar.

In order to investigate the disposition of the lipid modification of tirzepatide within the peptide-receptor complexes, we modeled in the C20 diacid conjugate, the Ct, and other unresolved atoms. A series of 1-µsec molecular dynamics (MD) explicit solvent simulations were then performed on models of tirzepatide in complex with either the GIPR or the GLP-1R in the membrane with the heterotrimeric G protein. The results revealed that the linker–fatty acid chain exists in multiple conformations (Fig. 3). Moreover, in the MD analyses, the lipid moiety makes a relatively low number of specific and persistent interactions with either receptor (*Right* panel of Fig. 3*B*). MD also indicated that an intramolecule hydrogen bond between the lipid chain and glutamine at position 24 of tirzepatide is more prevalent with the GIPR (∼40% of the simulation time) than in the GLP-1R complex (∼18% of the simulation time, *Left* panel of Fig. 3*B*). Overall, intermittent hydrogen bonding of the lipid moiety with the receptor is more distributed and prevalent in the GIPR than in the GLP-1R (*Right* panel of Fig. 3*B*). Consistent with these observations, the diacid chain in the GIPR/tirzepatide complex was found to be more compact than in the bound GLP-1R complex, as indicated by its mean radius of gyration (6.8 Å for the GIPR versus 7.6 Å for the GLP-1R) (Fig. 3*C*), while maintaining similar distributions for solvent accessible surface area and polar surface area of the lipid moiety (*SI Appendix*, Fig. S8). MD snapshots (Fig. 3*D*) demonstrate conformational diversity of tirzepatide in both receptors.

### Pharmacological Basis for the Function of Tirzepatide.

From the analyses of the cryo-EM structures of tirzepatide in complex with both receptors and the hypotheses proposed upon performing the MD simulations, a series of peptide analogs was synthesized to investigate the molecular basis underlying the pharmacological activity of tirzepatide. This was a knowledge-based design approach that deconstructed tirzepatide to evaluate its receptor binding and signaling properties. We note that the albumin-binding propensity of fatty acid-modified peptides can confound a pharmacological comparison of molecules, given the common use of albumin and serum as nonspecific blocking agents in biological assays. Therefore, to obviate this, entirely albumin-free assays supplemented with bovine casein or bacitracin were used to prevent nonspecific binding (10).

Competition binding assays revealed that removal of the lipidic side chain (TZP^ΔC20^) yields a ligand with approximately fourfold greater affinity than tirzepatide at the GIPR (Fig. 4*A*). The fatty acid modification feature of tirzepatide is necessary for the sustained pharmacokinetics of the molecule (1). The binding assay results indicate that a higher affinity parent peptide is an important contributor to the overall affinity of tirzepatide because it allows the addition of the fatty acid moiety that ultimately results in tirzepatide having equal affinity to that of GIP, a characteristic needed for its imbalanced potency pharmacology. Similar to the effect on affinity, removal of the lipid improved the potency of the ligand for stimulating GIPR-mediated activation of Gs (Fig. 4*B*) and receptor-induced accumulation of intracellular cAMP (Fig. 4*C*). Consistent with the therapeutic aim of a dual GIP/GLP-1 receptor agonist possessing activity at the GIPR that is similar to GIP, the amino acid sequence of tirzepatide contains many of the same residues that are found in GIP, especially those located within the N-terminal half of the ligand that make critical interactions with the GIPR binding pocket (Fig. 1). In particular, examination of the cryo-EM structures revealed the potential importance of the N-terminal tyrosine of both GIP and tirzepatide in interacting deep near the bottom of the receptor core (Fig. 1). In agreement with this model, changing tyrosine to histidine (TZP^ΔC20,Y1H^), to match the first position of GLP-1, weakened the binding affinity by ∼20-fold (Fig. 4*A*). Similarly, this substitution also decreased efficacy for Gs activation and cAMP accumulation (Fig. 4 *B* and *C*). These results point to the importance of the native tyrosine at this position for maintaining GIPR affinity and full agonism.

**Fig. 5.**
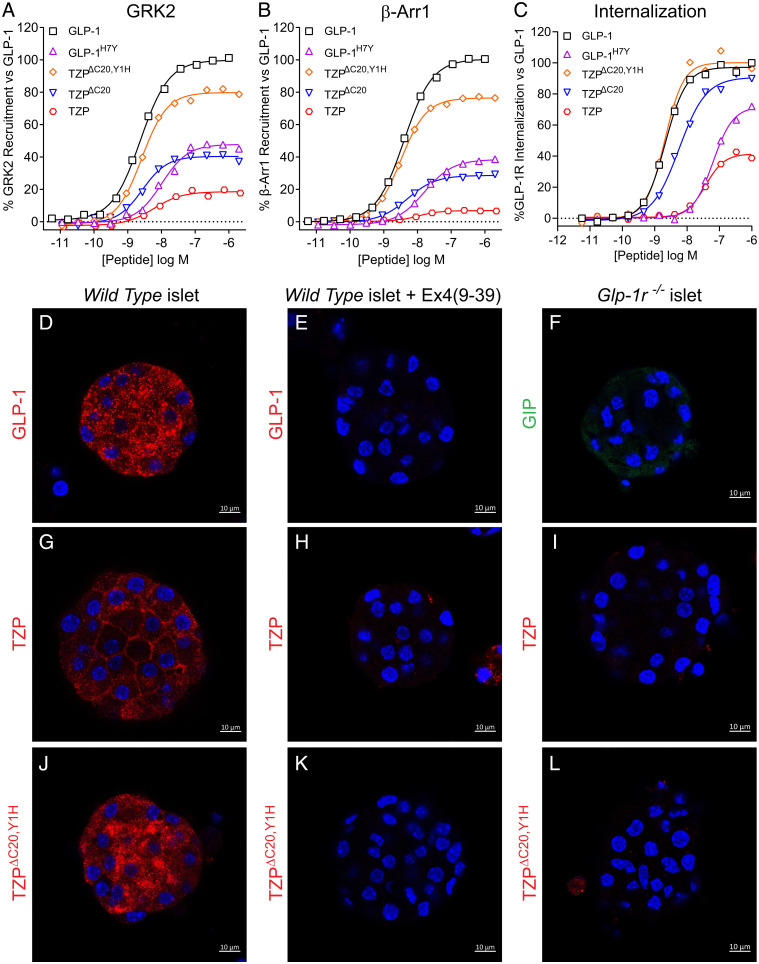
Biased pharmacology of TZP at the GLP-1R occurs through a composite effect on signaling efficacy by the N-terminal tyrosine and the C20 diacid fatty acid moiety. Mechanistic pharmacology studies investigating receptor binding and signal transduction were performed using TZP, an analog thereof lacking the lipid moiety (TZP^ΔC20^), and a derivative that also contains histidine in place of tyrosine at position 1 (TZP^ΔC20,Y1H^). Non-G protein signaling by TZP at the GLP-1R was investigated using assays for GRK2 (*A*) and β-arrestin (*B*) recruitment. In both systems, TZP is shown to be a weak, partial agonist in comparison to GLP-1. Removal of the C20 diacid fatty acid moiety improved the responses, and the absence of the lipid moiety in combination with replacing tyrosine with histidine resulted in activity that is nearly fully efficacious. (*C*) Ligand-induced internalization of the GLP-1R was assessed using changes in the cell surface presentation of SNAP-tagged receptor in HEK293 cells. Relative to GLP-1, TZP is shown to be a weak, partial agonist at inducing internalization of the GLP-1R. Consistent with restoring β-arrestin recruitment, the TZP^ΔC20^ and TZP^ΔC20,Y1H^ derivatives are shown to proportionally improve ligand-induced receptor internalization. A derivative of glucagon-like peptide-1 containing tyrosine in place of histidine at position 7 (GLP-1^H7Y^) was used as a control. Data presented are representative of *n* ≥ 3 independent experiments. Summarized data are shown in *SI Appendix*, Table 2. (*D–L*) Representative confocal images of pancreatic islets labeled with fluorescently tagged GLP-1, TZP, TZP^ΔC20,Y1H^, or GIP. Red Fluorescence (or green fluorescence for GIP) was detected following incubation of islets from wild-type (*D*, *G*, *J*) or *Glp-1r* null (*F*, *I*, *L*) mice with 30 nM of GLP-1^AF647^, TZP^AF647^, TZP^ΔC20,Y1H-AF647^, or (D-Ala2)GIP^AF488^ for 30 min. (*E*, *H*, and *K*) An additional set of islets from wild-type mice were preincubated with 2 µM GLP-1R antagonist exendin-4_(9-39)_ prior to treatment with the fluorescently labeled ligands. Nuclei are stained in blue with Hoechst 33342. log M; log Molar. (Scale bars, 10 µm.)

By contrast with tirzepatide mimicking the actions of GIP, its pharmacology at the GLP-1R differs from that of GLP-1. Previous studies showed that the affinity of tirzepatide for binding the GLP-1R is ∼fivefold weaker than that of GLP-1, manifesting in weaker potency and reduced efficacy in stimulating Gs activation, decreased potency in cAMP signaling, and little ligand-induced β-arrestin recruitment (10). From a therapeutic standpoint, the bias toward cAMP signaling versus β-arrestin recruitment may be advantageous for the GLP-1 component of tirzepatide as it fosters less agonist-induced desensitization. The findings in this current report further highlight the pharmacological differences of tirzepatide at the GLP-1R versus the GIPR. For instance, as opposed to the increase in affinity for the GIPR that occurred upon removal of the lipid, the affinity of tirzepatide for binding the GLP-1R was unaffected by the loss of the side chain (Fig. 4*D*). However, the nonacylated analog displayed higher efficacy in GLP-1R-stimulated Gs activation (Fig. 4*E*) and stronger potency in cAMP accumulation (Fig. 4*F*). Additionally, changing the N-terminal tyrosine to histidine in the nonacylated analog further improved potency, resulting in a ligand that is close to functionally equivalent to GLP-1 in these assays (Fig. 4 *E* and *F*). Although the influence of the noncognate tyrosine in combination with the presence of the acyl modification reduces the efficacy of GLP-1R-stimulated Gs signaling, this pharmacological profile remains sufficient to fully enhance insulin secretion and reduce hyperglycemia through the GLP-1R, as previously demonstrated by tirzepatide in cultures of pancreatic islets and glucose tolerance tests of *Gipr* null mice (1).

For class B G protein coupled receptors (GPCRs), including the GIPR and the GLP-1R, the N-terminal ECD is a unique structural feature that is implicated in the mechanism of ligand recognition. Interestingly, during the processing of the GLP-1R/tirzepatide cryo-EM data, three-dimensional (3D) classification revealed a unique class of GLP-1R/G protein complexes that showed no density for tirzepatide, a totally disordered ECD, and a wide-open extracellular pocket of the TM domain (*SI Appendix*, Figs. S2 and S9). Given that tirzepatide has nanomolar affinity for binding the full-length GLP-1R (Fig. 4*D*), and a saturating amount of tirzepatide was added during the sample preparation, the presence of apo complexes points to the hypothesis that tirzepatide engages with the GLP-1R TM bundle less stably than the ECD, consistent with the two-step model proposed for class B GPCRs (31). Therefore, to directly assess the contribution of ECD binding to ligand affinity, we purified the ECDs of both receptors and measured direct binding of peptide ligands using surface plasmon resonance. Ligands were found to bind the ECD of the GIPR with affinities in the low micromolar range, specifically tirzepatide (equilibrium constant [K_D_] = 4.2 µM) and tirzepatide^ΔC20^ (K_D_ = 1.7 µM) indicating that the enhanced receptor affinity of tirzepatide^ΔC20^ is principally independent of ECD binding for the GIPR (*SI Appendix*, Fig. S10). By contrast, binding to the GLP-1R ECD was of substantially higher affinity for tirzepatide (K_D_ = 23 nM) versus tirzepatide^ΔC20^ (K_D_ = 111 nM) (*SI Appendix*, Fig. S10), suggesting that the enhanced affinity delivered by the lipid moiety may be accounted for by interactions with the ECD of the GLP-1R.

Assays of non-G protein signaling were then used to further investigate the divergent pharmacology of tirzepatide at the GLP-1R. In line with previous results showing low efficacy/partial agonism of tirzepatide-induced recruitment of β-arrestin to the GLP-1R (10), tirzepatide demonstrated a similar profile in recruiting G protein-coupled receptor kinase 2 (GRK2) (Fig. 5 *A* and *B*), which helps terminate signaling by phosphorylating the receptor to enable binding of β-arrestins (32). Studies of the tirzepatide analogs in both assays revealed a modest increase in recruitment efficacy of the nonacylated parent peptide, and nearly full efficacy was achieved with the ligand that also contains the tyrosine-to-histidine replacement (Fig. 5 *A* and *B*). Since agonist-induced receptor internalization is often mediated by β-arrestin trafficking, GLP-1R internalization by the analogs was assessed using the N terminus SNAP-tag system. In these experiments, the potency and efficacy of tirzepatide to induce GLP-1R internalization were both greatly increased in the absence of the lipid (Fig. 5*C*). The ligand with the histidine at position one (TZP^ΔC20,Y1H^) showed a further slight improvement, resulting in equipotency to GLP-1 in the assay (Fig. 5*C*).

To extend these findings beyond the heterologous cellular systems, fluorescently labeled peptides were synthesized and used in orthogonal experiments to visualize ligand-induced receptor internalization in pancreatic islets. In these studies, GLP-1^AF647^, tirzepatide^AF647^, tirzepatide^ΔC20,Y1H-AF647^, or (D-Ala2)GIPR^AF488^ were incubated in static cultures of islets isolated from wild-type or *Glp-1r* null mice. Imaging by confocal microscopy showed that treatment with labeled GLP-1 (Fig. 5*D*) or tirzepatide^ΔC20,Y1H^ (Fig. 5*J*) resulted in a portion of the ligand appearing intracellularly as punctate fluorescent signal accumulated in the cytoplasm and perinuclear regions of islet cells. Intracellular fluorescence intensities for labeled GLP-1 and tirzepatide^ΔC20,Y1H^ were 104.5 ± 7.5 SEM (*n* = 22) and 85.3 ± 5.9 SEM (*n* = 16), respectively. However, the intracellular fluorescence intensity in islets incubated with tirzepatide^AF647^ was only 46.0 ± 3.6 SEM (*n* = 30) (Fig. 5*G*). As controls, islets pretreated with the GLP-1R antagonist exendin-4_(9-39)_ (Fig. 5 *E*, *H*, and *K*) or from *Glp-1r* null mice (Fig. 5 *F*, *I*, and *L*) showed negligible intracellular fluorescence (intensities of ≤4.0 at *n* ≥ 13), supporting a mechanism of GLP-1R-mediated ligand internalization. Of note, fluorescently tagged semaglutide showed intracellular staining similar to that of labeled GLP-1 in treated islets (*SI Appendix*, Fig. S11), supporting the notion that lipid modification in general does not prevent ligand internalization. Also, we speculate that a lack of apparent GIPR-mediated ligand internalization is due to differences in the magnitude of GIP internalization, as has been previously reported (10, 33), and/or the sensitivity of the method. Overall, the limited ability of tirzepatide to cause GLP-1R desensitization through GRK2/β-arrestin recruitment is consistent with its inability to induce GLP-1R internalization.

Together, the studies in this report investigated the molecular mechanism and structural determinants of the unique pharmacological profile of tirzepatide. Summation of the overall findings points to a model whereby biased agonism at the GLP-1R is driven by multiple mechanisms. Key interactions with the N terminus of tirzepatide are implicated in coordinating allosteric transitions essential for receptor activation, as is the case for other biased ligands (24, 29, 34). Consistent with prior studies (35, 36), we observed that the exendin-4 homologous sequence in the C terminus of tirzepatide facilitates a high-affinity interaction with the GLP-1R relative to the GIPR. By the nature of the classic two-step mechanism for class B peptide binding (31), the ECD-driven affinity of tirzepatide may engender a pharmacological opportunity for N-terminal residue modification while maintaining full-length receptor binding affinity. Modification of the N terminus of canonical class B GPCR ligands is well demonstrated to alter transducer efficacy (14, 37–39). Recent insights into class B GPCR activation suggest a complex, multistep mechanism for activation of heterotrimeric G proteins (40, 41), but the mechanism is less clear with respect to β-arrestin recruitment. We have previously observed that tirzepatide exhibits partial agonism for G protein activation at the GLP-1R (10), and this may represent a central mechanism of biased agonist pharmacology.

In conclusion, peptide therapeutics represent an increasingly important modality for drug discovery (42). Often, fatty acid modification is utilized as an effective approach for enhancing the in vivo half-life of peptides. This occurs in large part because binding of acylated peptides to serum albumin shields them from proteolysis and excretion. We demonstrate that peptide structure activity–relationships (SAR) are influenced/determined by acylation in a receptor and signal transduction dependent manner. As such, the pharmacological profile of tirzepatide is combinatorially determined by both the peptide sequence and the presence of the lipidic moiety. Uniquely, the lipid modification of tirzepatide in combination with tyrosine 1 is a key feature for the reduced efficacy of tirzepatide for GRK2 and β-arrestin recruitment and consequently internalization by the GLP-1R. Surprisingly, the effects of acylation are differential in that the nonpeptidic portion of the molecule reduces GIPR affinity but diminishes GLP-1R efficacy. The realization that SAR for both peptide and lipid modifications can drive subtle but potentially significant pharmacological effects (partial or biased agonism) suggests that the design of new therapeutics should encompass a wider swath of pharmacological, structural, and computational approaches to understand the complex interplay between receptors and acyl-peptide drugs.

## Methods

### Ligands.

All peptides were synthesized at Eli Lilly and Company using a standard solid-phase peptide synthesis methodology employing an Fmoc protecting group strategy, using Rink-amide resins with a loading of 0.35 to 0.88 mmol/g. Analogs containing linker–fatty acid modifications were accessed by incorporation of an Fmoc-l-Lys(Mtt)-OH residue at the appropriate position, in tandem with Boc-protection of the N-terminal residue. Orthogonal removal of the Mtt protecting group was achieved through treatment with 30% hexafluoroisopropanol in dichloromethane, prior to stepwise synthesis of the linker–fatty acid group with standard Fmoc chemistry. Following completion of the synthesis, peptides were cleaved from the resin by treatment with Trifluoroacetic acid (TFA)/water/triisopropylsilane/1,2-ethanedithiol (EDT) (85:5:5:5) for 2 h, followed by precipitation and washing with cold ether. Peptides were purified by reverse-phase high-performance liquid chromatography (RP-HPLC) using gradients of acetonitrile and water containing 0.1% TFA, to ≥95% purity. Selected analogs were fluorescently labeled at their C termini, first by incorporation of an additional C-terminal Fmoc-l-Lys(Mtt)-OH residue, which was similarly deprotected and functionalized with propargyl-PEG2-acid (Broadpharm), prior to synthesis of the remaining sequence and purification as described above. The resulting alkyne-tagged peptide precursors were then conjugated to AFDye 647 Azide (Click Chemistry Tools) or AFDye 488 Azide (Sigma Aldrich) using Cu-catalyzed azide-alkyne cycloaddition chemistry in water/dimethylformamide (1:1) and repurified by RP-HPLC. The positive allosteric modulators (PAMs) are as follows: LSN3451217 (4-[(3*S*)-2-[(2-hydroxy-4-isopropyl-phenyl)methyl]-6-methoxy-3-(methoxymethyl)-3,4-dihydro-1H-isoquinolin-5-yl]-2-methyl-phenol, hydrochloride salt) and LSN3556672 (2-fluoro-6-(isobutylamino)-4-[(1*R*)-2-[(1*S*)-1-(4-isopropylphenyl)ethyl]-6-methoxy-1-methyl-3,4-dihydro-1H-isoquinolin-5-yl]phenol, dihydrochloride salt). These compounds are modulators of the GLP-1R and GIPR, respectively, and were used to help form stable complexes of tirzepatide with the receptors.

### Constructs and Insect Cell Expression.

The human GIPR (residues 24 to 466) and GLP-1R (residues 24 to 422) cDNAs were subcloned into the pFastBac1 vector containing an N-terminal FLAG tag and a 3C protease site. Sf9 cells were infected with P2 virus produced according to the manufacturer’s protocol to produce GIPR- and GLP-1R-expressing cell pellets. The human Gsα subunit was modified with its 1 to 25 residues being replaced by 1 to 18 residues of the human Gαi at its N terminus to allow the binding of a single-chain variable fragment scFv16 as a stabilizing partner (43). This construct, named GsαiN18, was cloned into pVL1392. Human Gβ1 and Gγ2 were cloned into pFastBac Dual on the same vector. The P2 virus stocks were produced according to the manufacturer’s protocol. Hi-5 cells were infected with both GsαiN18 and Gβ1-Gγ2 viruses to produce the heterotrimeric GsiN18 protein cell pellets.

### Complex Formation and Purification.

The GIPR/GIP complex and the GIPR/tirzepatide and GLP-1R/tirzepatide complexes were formed and purified with similar strategies and are therefore described together. Steps and details that are specific to an individual sample are noted. Heterotrimeric GsiN18 was purified by Ni-affinity column and ion-exchange chromatography (44). scFv16 and Nb35 were produced as described previously (43, 44). Receptor/G protein complexes (referred to as the complex(es) in the following text) were formed in membrane. To prepare the complex samples, pellets of Sf9 cells expressing the respective receptor were lysed, and membrane samples were collected and washed. The complexes were formed by incubating purified molar excesses of heterotrimeric GsiN18, Nb35, scFv16, and 10 µM of the respective peptide (GIP or tirzepatide) with membranes overnight. For the GIPR/tirzepatide and GLP-1R/tirzepatide samples, 10 µM LSN3556672 and LSN3451217 were added, respectively. Samples were solubilized in buffer comprised of 1% DDM, 0.5% 3-[(3-Cholamidopropyl)dimethylammonio]-1-propanesulfonate hydrate, 0.2% cholesteryl hemisuccinate (CHS), 30 mM Hepes (pH 7.8), 150 mM NaCl, 30% glycerol, 25 µM tris(2-carboxyethyl)phosphine, 2.5 mg/mL leupeptin, 0.16 mg/mL benzamidine, 1 µM peptide, and 2 µM of the respective PAM for the GIPR/tirzepatide and GLP-1R/tirzepatide sample. Complexes were then purified by affinity chromatography using anti-FLAG M1 resin. For GIPR/GIP and GIPR/tirzepatide purification, the resin was washed with buffers containing high (1% DDM, 0.5% CHAPS, 0.2% CHS) and low (0.1% DDM, 0.05% CHAPS, 0.02% CHS) detergent concentrations, alternatively. Other components of the buffer were the same, as follows: 30 mM Hepes (pH 7.8), 150 mM NaCl, 10% glycerol, 25 µM TCEP, 1 µM tirzepatide, and 2 µM of the PAM. The volume used was 12 column volumes for each buffer. For the GLP-1R/tirzepatide sample, the resin was washed with 10 column volumes of 0.1% DDM, 0.05% CHAPS, 0.02% CHS, 30 mM Hepes pH 7.8, 150 mM NaCl, 10% glycerol, 25 µM TCEP, 1 µM tirzepatide, and 2 µM of the PAM. After washing, the sample was exchanged in a step-wise manner in buffer comprised of 30 mM Hepes (pH 7.5), 150 mM NaCl, 2.5 mM CaCl2, 1 µM of the respective peptide, 2 µM of the respective PAM, 25 μM TCEP, 0.25% lauryl maltose neopentyl glycol (MNG; NG310 Anatrace), 0.25% GDN101 (Anatrace), 0.048% 1-palmitoyl-2-oleoyl-sn-glycero-3-phospho-1’-rac-glycerol (POPG; Avanti), and 0.03% cholesterol (Sigma-Aldrich). Complexes were eluted and further purified by size-exclusion chromatography using a Superdex S200 10/300 GL column with running buffer of 30 mM Hepes (pH 7.5), 150 mM NaCl, 1 µM of the respective peptide and 2 µM of the respective PAM, 100 μM TCEP, 0.015% MNG, 0.005% GDN101, 0.00192% POPG, and 0.0012% cholesterol. The fractions for monomeric complexes were collected and concentrated individually for electron microscopy experiments.

### Cryo-EM Data Acquisition and Processing.

Samples of 3.5 µL of purified complexes at a concentration of ∼10 mg/mL were applied to glow-discharged holey carbon grids (Quantifoil R1.2/1.3, 200 mesh) and subsequently vitrified using a Vitrobot Mark IV (Thermo Fisher Scientific). Specimens were visualized using a Titan Krios electron microscope (Thermo Fisher Scientific) with an energy filter operating at 300-kV accelerating voltage using a K3 Summit direct electron detector (Gatan, Inc.) in counting mode. Statistics for the data collection are indicated in *SI Appendix*, Table S1.

Data processing was performed in Relion3.1 (45). Dose-fractionated image stacks were subjected to beam-induced motion correction using MotionCor2 (46). Contrast transfer function (CTF) parameters for each micrograph were determined by Gctf (47). Manual selection of micrographs based on their quality and CTF estimated resolution was performed prior to subsequent steps. Particle selection with two-dimensional (2D) and 3D classifications were performed on a binned dataset with a pixel size of 1.64 Å. Semiautomated particle selection was carried out using a template from a previously reported GLP-1R/GLP-1/Gs/Nb35 complex (16); these particles were subjected to 2D and 3D classification. After 3D classification, classes of particles that generated the best 3D maps were selected, and unbinned versions of these particles were used as the input for 3D autorefinement. Bayesian polishing and CTF refinement (48) were applied to further improve the map. The flow charts for individual samples are shown in *SI Appendix*, Figs. S1 and S2. Reported resolution is based on the gold-standard Fourier shell correlation using the 0.143 criterion (*SI Appendix*, Figs. S1 and S2).

### Model Building and Refinement.

Initial model building was done using by rigid-body fitting of the reported GLP-1R/GLP-1/Gs/Nb35 structure (RCSB Protein Data Bank code 6VCB). The sequence was mutated to match GIPR, GIP, or tirzepatide when applicable in respective models. The starting model was then subjected to iterative rounds of manual and real space refinement in Coot (49, 50) and Phenix (51), respectively. Final models were visually inspected for general fit to the maps, and geometry was further evaluated using Molprobity (52). Final refinement statistics for the models are summarized in *SI Appendix*, Table S1.

Atomic coordinates and the cryo-EM density maps have been deposited in the Protein Data Bank (PDB) and The Electron Microscopy Data Bank (EMDB). The accession numbers are as follows: GIPR/GIP (PDB: 7RA3; EMDB: EMD-24334), GIPR/tirzepatide (PDB: 7RBT; EMDB: EMD-24401), GLP-1R/tirzepatide (PDB: 7RGP; EMDB: EMD-24453), and GLP-1R apo form (PDB: 7RG9; EMDB: EMD-24445).

### MD Simulations.

Missing receptor loops and side chains were added to both cryo-EM structures of the tirzepatide:GIPR:G protein and tirzepatide:GLP-1R:G protein complexes reported herein, using the homology modeling process and 3D and peptide builders in MOE (Molecular Operating Environment). The linker–fatty acid lipid chain conjugated to Lys20^Tzp^ and the C-terminal 33 to 39 residues, which were not observed in the cryo-EM structures, were modeled in extended conformations and placed away from the receptors as the initial structure models in order to avoid introducing artificial biased interactions with the receptors. The complex structures were further energy minimized with restrained heavy atoms using Schrodinger software (version 202.3). The transferable intermolecular potential with 3 points water and the phosphatidylcholine membrane were then added to the complex systems for running MD. The membrane positions were set to the receptor residues 136 to 159, 172 to 193, 215 to 239, 258 to 280, 294 to 318, 339 to 362, and 372 to 395 in GIPR; and 143 to 165, 179 to 200, 225 to 249, 268 to 290, 304 to 329, 350 to 373, and 381 to 405 in GLP-1R. The alpha carbon atoms of the TM domain and intracellular G protein components were harmonically restrained (0.1 kcal/(mol.A^2^)), with the ECDs of the receptors, the peptides, and the modulators unrestrained. Systems contained 220K atoms, and the simulations were run with Desmond MD package using the OPLS3e force field (53). An analysis was performed dismissing the first 10 nsec of the trajectory. The radius of gyration, hydrogen bonds, and polar surface area were monitored during the simulations. All MD simulations and analyses were carried out on V100 Nvidia graphics processing unit using Schrodinger software (version 2020.3).

### In Vitro Pharmacology.

Recombinant human GIPR and GLP-1R expressing cell lines, cAMP accumulation assays, [^35^S]GTPγS binding assays, NanoBRET β-arrestin 1 recruitment, and [^125^I]-GLP-1(7-36)NH_2_ and [^125^I]-GIP (1–42) radioligand binding studies were conducted exactly as described in reference 10.

GRK2 recruitment to the GLP-1R was quantified using by the NanoBRET method (54). pcDNA3.1-based vectors encoding NanoLuc fused to the N terminus of GRK2 (NP_001610) and a C-terminal fusion of GLP-1R and the HaloTag were used to transfect freestyle HEK cells (55). After 48 h, transfected cells were incubated in phosphate-buffered saline (PBS) containing 0.1% (wt/vol) bovine casein and varying concentrations of the ligands. Bioluminescence resonance energy transfer (BRET) was initiated using the Nano-Glo substrate as donor and the NanoBRET 618 ligand as acceptor. Emission was measured at 460 nm and 610 nm, and the BRET ratio was calculated. The ligand-induced interaction of the fusion proteins as a percentage of maximal GLP-1 was plotted versus the concentration of ligand.

To assess ligand-induced receptor internalization, HEK293 cells expressing SNAP-GLP-1R were labeled with 100 nM Tag-Lite SNAP-Lumi4-Tb (donor), washed, and incubated in Opti-MEM containing 100 µM fluorescein-O’ acetic acid (acceptor). Varying concentrations of the ligands were then added, and mixtures were incubated at 5% CO_2_/37 °C. Data were collected using an EnVision plate reader. Percent internalization compared to maximal GLP-1 was plotted versus the concentration of ligand. Additional experimental details for the above procedures were previously reported (10). Data for all assays were fit to the four-parameter logistic model in Genedata Screener 17 or GraphPad Prism 9 software.

### ECD Binding Assay.

The isolated ECDs of human GIPR (amino acids 26 to 138) and GLP-1R (amino acids 24 to 145) were expressed as 6His-tagged secreted proteins in CHO cells. DNA encoding each protein was transfected into CHO cells for expression. After expression, the proteins were purified by affinity chromatography (HIS-Trap Ni, Cytiva) followed by size exclusion chromatography using a HiLoad 26/600 Superdex 75pg column (Cytiva) with PBS buffer. Fractions were analyzed by sodium dodecyl-sulfate polyacrylamide gel electrophoresis, and fractions meeting purification criteria were pooled, then 0.22 μm filtered. Surface plasmon resonance measurements were made using a Biacore T200 (Cytiva) and analyzed using T200 Evaluation Software Version 3.1. Receptor ectodomain proteins were covalently immobilized (∼250 resonance units) on Sensor Chip CM4 BR100534 using the Amine coupling Immobilization Wizard in the Biacore T200 Control Software Version 2.0.2. Running buffer was HBS-EP (10 mM Hepes, 150 mM NaCl, 3 mM EDTA, 0.05% P20 [pH 7.6]; Teknova). Concentration series for each sample were made by serial dilution in running buffer. The samples were injected for 150 sec at a 30-µL/min flow rate, followed by 300 sec of dissociation. The surface was regenerated between samples with 10 mM glycine (pH 1.7).

### Labeling of Pancreatic Islets.

Mouse and rat islets were isolated as previously described (56), allowed to recover for 48 h in RPMI 1640 media (Gibco) containing 10% fetal bovine serum (FBS), and then adhered to imaging assay plates using Cell Tak cell adhesive (Corning) in RPMI 1640 media containing 0.5% FBS for 120 min at 5% CO_2_/37 °C. For receptor specificity, during the last hour of the adherence phase, a portion of islets were incubated with 2 µM exendin-4_(9-39)_ to assess GLP-1R binding. Islets were labeled with fluorescent ligands (10 to 30 nM) in assay buffer consisting of Hank’s balanced salt solution with 0.1% casein for 30 min at 5% CO_2_/37 °C. Background fluorescence was determined on islets incubated with assay buffer only. Following labeling, islets were rapidly washed two times with PBS to remove unbound fluorescence then immediately fixed in 4% paraformaldehyde, 0.1% Tween20, and 0.4 µg/mL Hoechst 33342 for 30 min at room temperature. The mouse islets were imaged using a Zeiss LSM800 confocal microscope with 63× oil objective. For the rat islets, after fixation, islets were stained with 125 nM AlexaFluor488 Phalloidin (Thermo Fisher Scientific) for 20 min at room temperature to detect the cell membrane. Then, images were taken with an Airyscan on the LSM800 and deconvoluted using Zen software (Blue edition 2.3 lite, Zeiss). Quantitation was also done using Zen software. The intracellular fluorescence intensity of individual islet cells was measured in the perinuclear region using a circular, 5-µm-diameter tool. The intensity values were corrected for background by subtracting fluorescence intensities recorded from vehicle-treated islets. Data were sampled from at least four individual cells from two to five individual islet images. Data are reported as mean ± SEM.

## Supplementary Material

Supplementary File

## Data Availability

Coordinates and cryo-EM maps data have been deposited in Protein Data Bank (PDB) and Electron Microscopy Data Bank (EMDB) (PDB: 7RA3, EMDB: EMD-24334) (PDB: 7RBT, EMDB: EMD-24401) (PDB:7RGP, EMDB: EMD-24453) (PDB: 7RG9, EMDB: EMD-24445).

## References

[1] T. Coskun , LY3298176, a novel dual GIP and GLP-1 receptor agonist for the treatment of type 2 diabetes mellitus: From discovery to clinical proof of concept. Mol. Metab. 18, 3–14 (2018). 30473097 10.1016/j.molmet.2018.09.009 PMC6308032

[2] K. B. Bokvist, T. Coskun, R. C. Cummins, J. Alsina-Fernandez, United States Patent No. US 9,474,780 B2. Available from: https://patentimages.storage.googleapis.com/e4/20/b1/04165a87d59f23/US9474780.pdf. (2016).

[3] R. J. Samms, M. P. Coghlan, K. W. Sloop, How may GIP enhance the therapeutic efficacy of GLP-1? Trends Endocrinol. Metab. 31, 410–421 (2020). 32396843 10.1016/j.tem.2020.02.006

[4] B. Finan , Reappraisal of GIP pharmacology for metabolic diseases. Trends Mol. Med. 22, 359–376 (2016). 27038883 10.1016/j.molmed.2016.03.005

[5] J. P. Frias , Efficacy and safety of LY3298176, a novel dual GIP and GLP-1 receptor agonist, in patients with type 2 diabetes: A randomised, placebo-controlled and active comparator-controlled phase 2 trial. Lancet 392, 2180–2193 (2018). 30293770 10.1016/S0140-6736(18)32260-8

[6] M. K. Thomas , Dual GIP and GLP-1 receptor agonist tirzepatide improves beta-cell function and insulin sensitivity in Type 2 diabetes. J. Clin. Endocrinol. Metab. 106, 388–396 (2021). 33236115 10.1210/clinem/dgaa863 PMC7823251

[7] J. M. Wilson , The dual glucose-dependent insulinotropic peptide and glucagon-like peptide-1 receptor agonist, tirzepatide, improves lipoprotein biomarkers associated with insulin resistance and cardiovascular risk in patients with type 2 diabetes. Diabetes Obes. Metab. 22, 2451–2459 (2020). 33462955 10.1111/dom.14174 PMC7756479

[8] J. P. Frías, Tirzepatide: A glucose-dependent insulinotropic polypeptide (GIP) and glucagon-like peptide-1 (GLP-1) dual agonist in development for the treatment of type 2 diabetes. Expert Rev. Endocrinol. Metab. 15, 379–394 (2020). 33030356 10.1080/17446651.2020.1830759

[9] J. P. Frías , Tirzepatide versus semaglutide once weekly in patients with Type 2 diabetes. N. Engl. J. Med. 385, 503–515. (2021). 34170647 10.1056/NEJMoa2107519

[10] F. S. Willard , Tirzepatide is an imbalanced and biased dual GIP and GLP-1 receptor agonist. JCI Insight 5, e140532 (2020).

[11] R. J. Samms , GIPR agonism mediates weight-independent insulin sensitization by tirzepatide in obese mice. J. Clin. Invest. 131, e146353 (2021).

[12] Q. Zhang , The glucose-dependent insulinotropic polypeptide (GIP) regulates body weight and food intake via CNS-GIPR signaling. Cell Metab. 33, 833–844.e5. (2021). 33571454 10.1016/j.cmet.2021.01.015 PMC8035082

[13] E. Yuliantie , Pharmacological characterization of mono-, dual- and tri-peptidic agonists at GIP and GLP-1 receptors. Biochem. Pharmacol. 177, 114001 (2020). 32360365 10.1016/j.bcp.2020.114001

[14] B. Jones , Targeting GLP-1 receptor trafficking to improve agonist efficacy. Nat. Commun. 9, 1602 (2018). 29686402 10.1038/s41467-018-03941-2 PMC5913239

[15] M. Lucey , Disconnect between signalling potency and in vivo efficacy of pharmacokinetically optimised biased glucagon-like peptide-1 receptor agonists. Mol. Metab. 37, 100991 (2020). 32278079 10.1016/j.molmet.2020.100991 PMC7262448

[16] Y. Zhang , Cryo-EM structure of the activated GLP-1 receptor in complex with a G protein. Nature 546, 248–253 (2017). 28538729 10.1038/nature22394 PMC5587415

[17] X. Zhang , Differential GLP-1R binding and activation by peptide and non-peptide agonists. Mol. Cell 80, 485–500.e7 (2020). 33027691 10.1016/j.molcel.2020.09.020

[18] H. Zhang , Structure of the glucagon receptor in complex with a glucagon analogue. Nature 553, 106–110 (2018). 29300013 10.1038/nature25153

[19] L. S. Hansen , N-terminally and C-terminally truncated forms of glucose-dependent insulinotropic polypeptide are high-affinity competitive antagonists of the human GIP receptor. Br. J. Pharmacol. 173, 826–838 (2016). 26572091 10.1111/bph.13384 PMC4761099

[20] M. B. N. Gabe, W. J. C. van der Velden, F. X. Smit, L. S. Gasbjerg, M. M. Rosenkilde, Molecular interactions of full-length and truncated GIP peptides with the GIP receptor—A comprehensive review. Peptides 125, 170224 (2020). 31809770 10.1016/j.peptides.2019.170224

[21] F. Zhao , Structural insights into hormone recognition by the human glucose-dependent insulinotropic polypeptide receptor. eLife 10, e68719 (2021). 34254582 10.7554/eLife.68719 PMC8298097

[22] C. Parthier , Crystal structure of the incretin-bound extracellular domain of a G protein-coupled receptor. Proc. Natl. Acad. Sci. U.S.A. 104, 13942–13947 (2007). 17715056 10.1073/pnas.0706404104 PMC1955799

[23] A. Qiao , Structural basis of G_s_ and G_i_ recognition by the human glucagon receptor. Science 367, 1346–1352 (2020). 32193322 10.1126/science.aaz5346

[24] Y. L. Liang , Phase-plate cryo-EM structure of a biased agonist-bound human GLP-1 receptor-Gs complex. Nature 555, 121–125 (2018). 29466332 10.1038/nature25773

[25] X. Zhang , Structure and dynamics of semaglutide- and taspoglutide-bound GLP-1R-Gs complexes. Cell Rep. 36, 109374 (2021). 34260945 10.1016/j.celrep.2021.109374

[26] D. Wootten , The extracellular surface of the GLP-1 receptor is a molecular trigger for biased agonism. Cell 165, 1632–1643 (2016). 27315480 10.1016/j.cell.2016.05.023 PMC4912689

[27] R. L. Dods, D. Donnelly, The peptide agonist-binding site of the glucagon-like peptide-1 (GLP-1) receptor based on site-directed mutagenesis and knowledge-based modelling. Biosci. Rep. 36, e00285 (2015). 26598711 10.1042/BSR20150253 PMC4718506

[28] G. Deganutti , Dynamics of GLP-1R peptide agonist engagement are correlated with kinetics of G protein activation. *Nat. Commun.* **13**, 92 (2021).

[29] T. Kawai , Structural basis for GLP-1 receptor activation by LY3502970, an orally active nonpeptide agonist. Proc. Natl. Acad. Sci. U.S.A. 117, 29959–29967 (2020). 33177239 10.1073/pnas.2014879117 PMC7703558

[30] J. Lau , Discovery of the once-weekly Glucagon-Like Peptide-1 (GLP-1) analogue semaglutide. J. Med. Chem. 58, 7370–7380 (2015). 26308095 10.1021/acs.jmedchem.5b00726

[31] S. R. Hoare, Mechanisms of peptide and nonpeptide ligand binding to Class B G-protein-coupled receptors. Drug Discov. Today 10, 417–427 (2005). 15808821 10.1016/S1359-6446(05)03370-2

[32] M. J. Lohse, J. L. Benovic, J. Codina, M. G. Caron, R. J. Lefkowitz, beta-Arrestin: A protein that regulates beta-adrenergic receptor function. Science 248, 1547–1550 (1990). 2163110 10.1126/science.2163110

[33] M. B. N. Gabe , Human GIP(3-30)NH_2_ inhibits G protein-dependent as well as G protein-independent signaling and is selective for the GIP receptor with high-affinity binding to primate but not rodent GIP receptors. Biochem. Pharmacol. 150, 97–107 (2018). 29378179 10.1016/j.bcp.2018.01.040

[34] H. Zhang , Autocrine selection of a GLP-1R G-protein biased agonist with potent antidiabetic effects. Nat. Commun. 6, 8918 (2015). 26621478 10.1038/ncomms9918 PMC4686834

[35] S. Runge , Differential structural properties of GLP-1 and exendin-4 determine their relative affinity for the GLP-1 receptor N-terminal extracellular domain. Biochemistry 46, 5830–5840 (2007). 17444618 10.1021/bi062309m

[36] R. López de Maturana, A. Willshaw, A. Kuntzsch, R. Rudolph, D. Donnelly, The isolated N-terminal domain of the glucagon-like peptide-1 (GLP-1) receptor binds exendin peptides with much higher affinity than GLP-1. J. Biol. Chem. 278, 10195–10200 (2003). 12524435 10.1074/jbc.M212147200

[37] B. Jones , Genetic and biased agonist-mediated reductions in beta-arrestin recruitment prolong cAMP signalling at glucagon family receptors. J. Biol. Chem. 296, 100133. (2020). 33268378 10.1074/jbc.RA120.016334 PMC7948418

[38] M. C. Lin, D. E. Wright, V. J. Hruby, M. Rodbell, Structure-function relationships in glucagon: Properties of highly purified des-His-1-, monoiodo-, and (des-Asn-28, Thr-29)(homoserine lactone-27)-glucagon. Biochemistry 14, 1559–1563 (1975). 164891 10.1021/bi00679a002

[39] J. Thulesen , The truncated metabolite GLP-2 (3-33) interacts with the GLP-2 receptor as a partial agonist. Regul. Pept. 103, 9–15 (2002). 11738243 10.1016/s0167-0115(01)00316-0

[40] T. M. Josephs , Structure and dynamics of the CGRP receptor in apo and peptide-bound forms. Science 372, eabf7258 (2021). 33602864 10.1126/science.abf7258

[41] D. Hilger , Structural insights into differences in G protein activation by family A and family B GPCRs. Science 369, eaba3373 (2020). 32732395 10.1126/science.aba3373 PMC7954662

[42] M. Muttenthaler, G. F. King, D. J. Adams, P. F. Alewood, Trends in peptide drug discovery. Nat. Rev. Drug Discov. 20, 309–325 (2021). 33536635 10.1038/s41573-020-00135-8

[43] S. Maeda , Development of an antibody fragment that stabilizes GPCR/G-protein complexes. Nat. Commun. 9, 3712 (2018). 30213947 10.1038/s41467-018-06002-w PMC6137068

[44] S. G. Rasmussen , Structure of a nanobody-stabilized active state of the β(2) adrenoceptor. Nature 469, 175–180 (2011). 21228869 10.1038/nature09648 PMC3058308

[45] S. H. Scheres, Processing of structurally heterogeneous cryo-EM data in RELION. Methods Enzymol. 579, 125–157 (2016). 27572726 10.1016/bs.mie.2016.04.012

[46] S. Q. Zheng , MotionCor2: Anisotropic correction of beam-induced motion for improved cryo-electron microscopy. Nat. Methods 14, 331–332 (2017). 28250466 10.1038/nmeth.4193 PMC5494038

[47] K. Zhang, Gctf: Real-time CTF determination and correction. J. Struct. Biol. 193, 1–12 (2016). 26592709 10.1016/j.jsb.2015.11.003 PMC4711343

[48] J. Zivanov , New tools for automated high-resolution cryo-EM structure determination in RELION-3. eLife 7, e42166 (2018). 30412051 10.7554/eLife.42166 PMC6250425

[49] P. Emsley, B. Lohkamp, W. G. Scott, K. Cowtan, Features and development of Coot. Acta Crystallogr. D Biol. Crystallogr. 66, 486–501 (2010). 20383002 10.1107/S0907444910007493 PMC2852313

[50] P. Emsley, K. Cowtan, Coot: Model-building tools for molecular graphics. Acta Crystallogr. D Biol. Crystallogr. 60, 2126–2132 (2004). 15572765 10.1107/S0907444904019158

[51] P. D. Adams , PHENIX: A comprehensive Python-based system for macromolecular structure solution. Acta Crystallogr. D Biol. Crystallogr. 66, 213–221 (2010). 20124702 10.1107/S0907444909052925 PMC2815670

[52] C. J. Williams , MolProbity: More and better reference data for improved all-atom structure validation. Protein Sci. 27, 293–315 (2018). 29067766 10.1002/pro.3330 PMC5734394

[53] K. Roos , OPLS3e: Extending force field coverage for drug-like small molecules. J. Chem. Theory Comput. 15, 1863–1874 (2019). 30768902 10.1021/acs.jctc.8b01026

[54] T. Machleidt , NanoBRET—A novel BRET platform for the analysis of protein-protein interactions. ACS Chem. Biol. 10, 1797–1804 (2015). 26006698 10.1021/acschembio.5b00143

[55] E. Lizano, J. L. Hayes, F. S. Willard, A synthetic method to assay adhesion-family G-protein coupled receptors. Determination of the G-protein coupling profile of ADGRG6(GPR126). Biochem. Biophys. Res. Commun. 534, 317–322 (2021). 33248691 10.1016/j.bbrc.2020.11.086

[56] A. B. Bueno , Positive Allosteric Modulation of the Glucagon-like Peptide-1 Receptor by Diverse Electrophiles. J. Biol. Chem. 291, 10700–10715 (2016). 26975372 10.1074/jbc.M115.696039 PMC4865917

