# Supplementary material for: Structural determinants of dual incretin receptor agonism by tirzepatide
Source: Proc Natl Acad Sci U S A. 2022 Mar 25;119(13):e2116506119. doi: 10.1073/pnas.2116506119 (PMC9060465; doi:10.1073/pnas.2116506119)
Supplement: Supplementary File [file pnas.2116506119.sapp.pdf]

PNAS
www.pnas.org

**Supplementary Information for**

# Structural determinants of dual incretin receptor agonism by tirzepatide

Bingfa Sun, Francis S. Willard, Dan Feng, Jorge Alsina-Fernandez, Qi Chen, Michal Vieth, Joseph D. Ho, Aaron D. Showalter, Cynthia Stutsman, Liyun Ding, Todd M. Suter, James D. Dunbar, John W. Carpenter, Faiz Ahmad Mohammed, Eitaro Aihara, Robert A. Brown, Ana B. Bueno, Paul J. Emmerson, Julie S. Moyers, Tong Sun Kobilka, Matthew P. Coghlan, Brian K. Kobilka, and Kyle W. Sloop

Brian K. Kobilka and Kyle W. Sloop
Email: kobilka@stanford.edu; sloop_kyle_w@lilly.com

**This PDF file includes:**

Figures S1 to S11
Tables S1 to S2

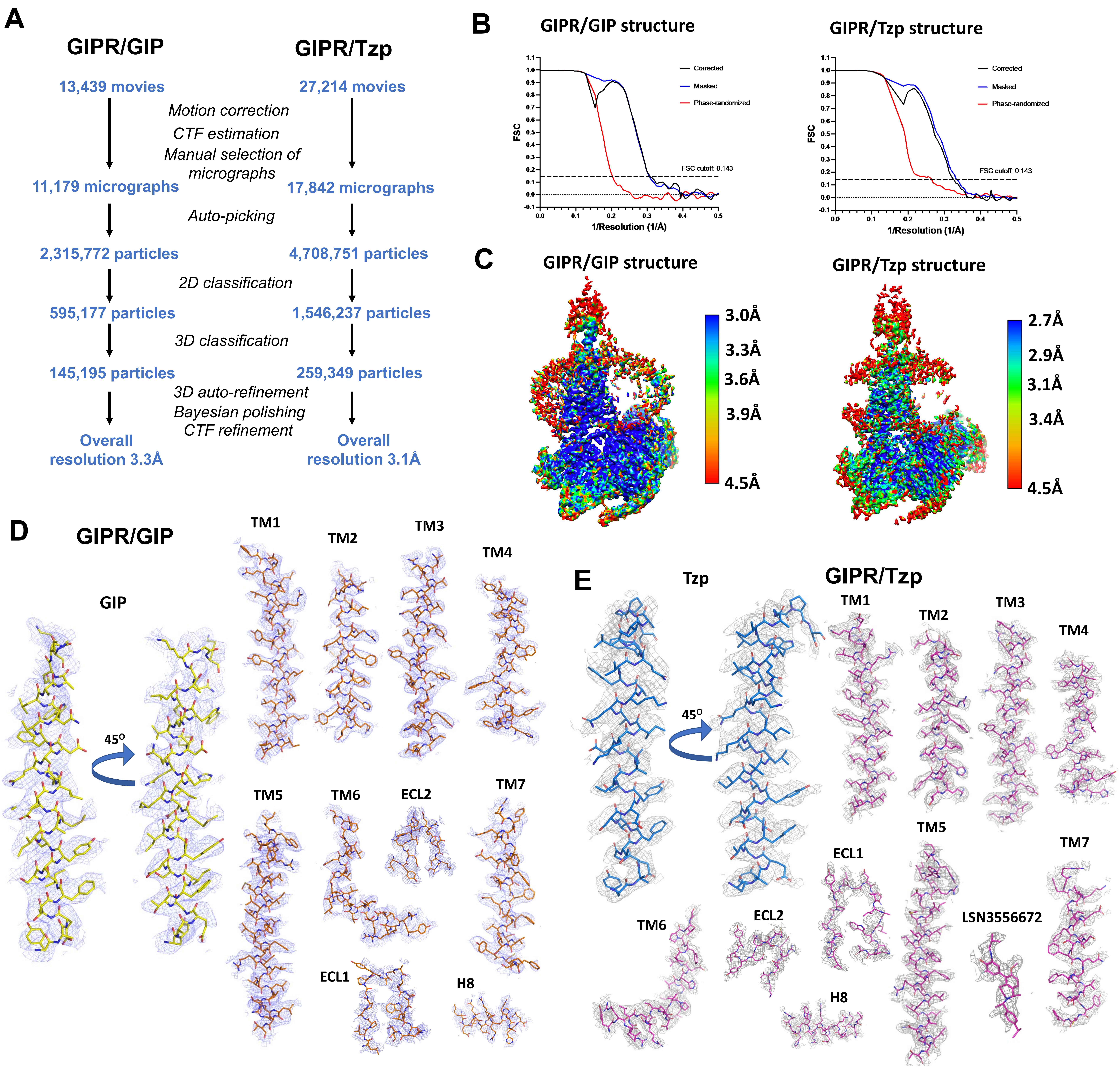

A
GIPR/GIP
GIPR/Tzp
13,439 movies
27,214 movies
Motion correction
CTF estimation
Manual selection of micrographs
11,179 micrographs
17,842 micrographs
Auto-picking
2,315,772 particles
4,708,751 particles
2D classification
595,177 particles
1,546,237 particles
3D classification
145,195 particles
259,349 particles
3D auto-refinement
Bayesian polishing
CTF refinement
Overall resolution 3.3Å
Overall resolution 3.1Å
B
GIPR/GIP structure
GIPR/Tzp structure
FSC
1/Resolution (1/Å)
Corrected
Masked
Phase-randomized
FSC cutoff: 0.143
C
GIPR/GIP structure
GIPR/Tzp structure
3.0Å
3.3Å
3.6Å
3.9Å
4.5Å
2.7Å
2.9Å
3.1Å
3.4Å
4.5Å
D
GIPR/GIP
GIP
45°
TM1
TM2
TM3
TM4
TM5
TM6
ECL2
TM7
ECL1
H8
E
Tzp
GIPR/Tzp
45°
TM1
TM2
TM3
TM4
TM5
TM7
ECL1
TM6
ECL2
LSN3556672
H8

**Fig. S1. Cryo-EM structure determination of GIPR/GIP and GIPR/tirzepatide structures.** (A) Cryo-EM data processing flow chart. (B) Gold standard Fourier shell correlation (FSC) curves of two individual half maps, indicating the global resolution at 0.143 FSC threshold. (C) Density map colored by local resolution. (D) The cryo-EM density map and model for GIPR/GIP structure are shown for GIP (zoomed in), all seven transmembrane (TM) helices, extracellular loops 1 and 2 (ECL1, ECL2), and helix 8 (H8) of the GIPR. (E) The cryo-EM density map and model for GIPR/Tzp structure are shown for Tzp (zoomed in), all seven TMs, ECL1, ECL2, and H8 of the GIPR.

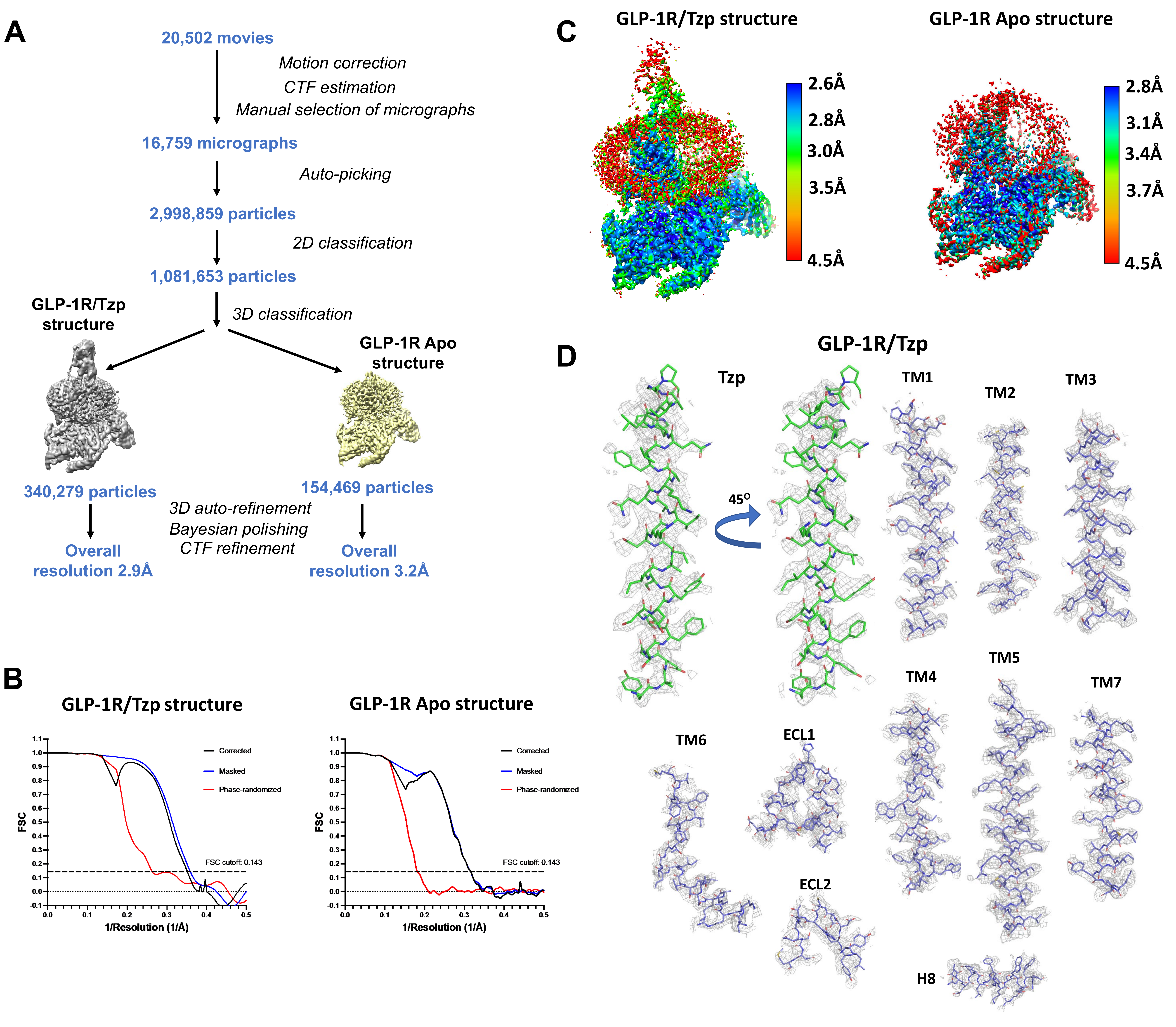
A
20,502 movies
Motion correction
CTF estimation
Manual selection of micrographs
16,759 micrographs
Auto-picking
2,998,859 particles
2D classification
1,081,653 particles
3D classification
GLP-1R/Tzp structure
GLP-1R Apo structure
340,279 particles
154,469 particles
3D auto-refinement
Bayesian polishing
CTF refinement
Overall resolution 2.9Å
Overall resolution 3.2Å
B
GLP-1R/Tzp structure
GLP-1R Apo structure
Corrected
Masked
Phase-randomized
FSC
FSC cutoff: 0.143
1/Resolution (1/Å)
C
GLP-1R/Tzp structure
GLP-1R Apo structure
2.6Å
2.8Å
3.0Å
3.5Å
4.5Å
2.8Å
3.1Å
3.4Å
3.7Å
4.5Å
D
GLP-1R/Tzp
Tzp
45°
TM1
TM2
TM3
TM4
TM5
TM7
TM6
ECL1
ECL2
H8

**Fig. S2. Cryo-EM structure determination of GLP-1R/tirzepatide structure.** (A) Cryo-EM data processing flow chart. At 3D classification, a population of particles showing no ECD and Tzp were separated, and a 3D reconstruction was performed to generate a map representing the GLP-1R apo-form (Apo) structure. (B) Gold standard Fourier shell correlation (FSC) curves of two individual half maps, indicating the global resolution at 0.143 FSC threshold. (C) Density map colored by local resolution. (D) The cryo-EM density map and model for GLP-1R/Tzp structure are shown for Tzp (zoomed in), all seven TMs, ECL1, ECL2, and H8 of the GLP-1R.

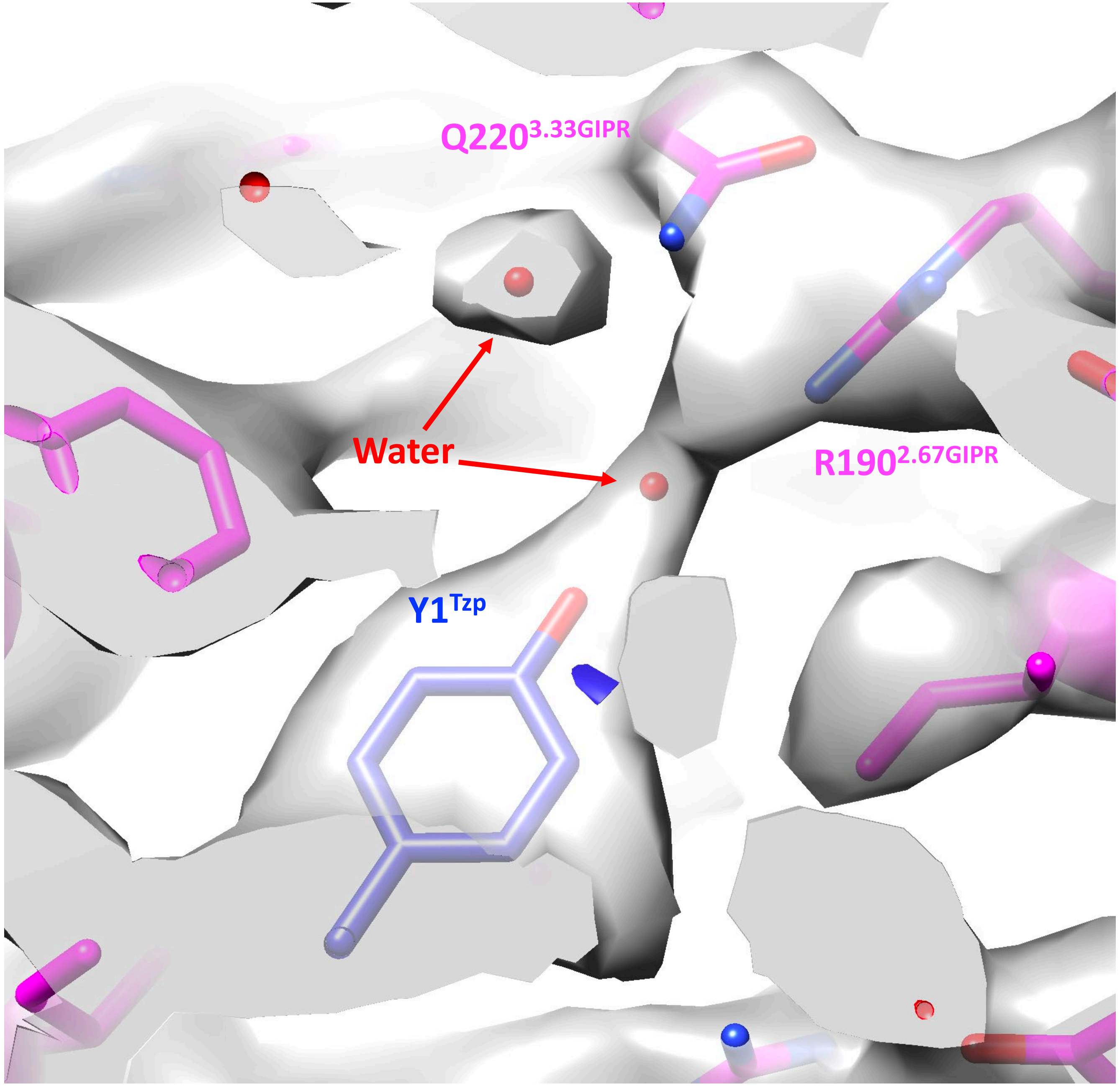
Q220^3.33GIPR
Water
R190^2.67GIPR
Y1^Tzp

**Fig. S3. Possible water mediated interactions between Tyr1$^{Tzp}$ and Arg190$^{2.67GIPR}$ and Gln220$^{3.33GIPR}$ are revealed in the GIPR/tirzepatide structure.** The cryo-EM map is shown in silver color. Residues of Tzp are colored in blue, and residues of GIPR are colored in magenta. Possible water molecules are shown in red spheres. The water molecules were not included in the coordinate file deposited to PDB.

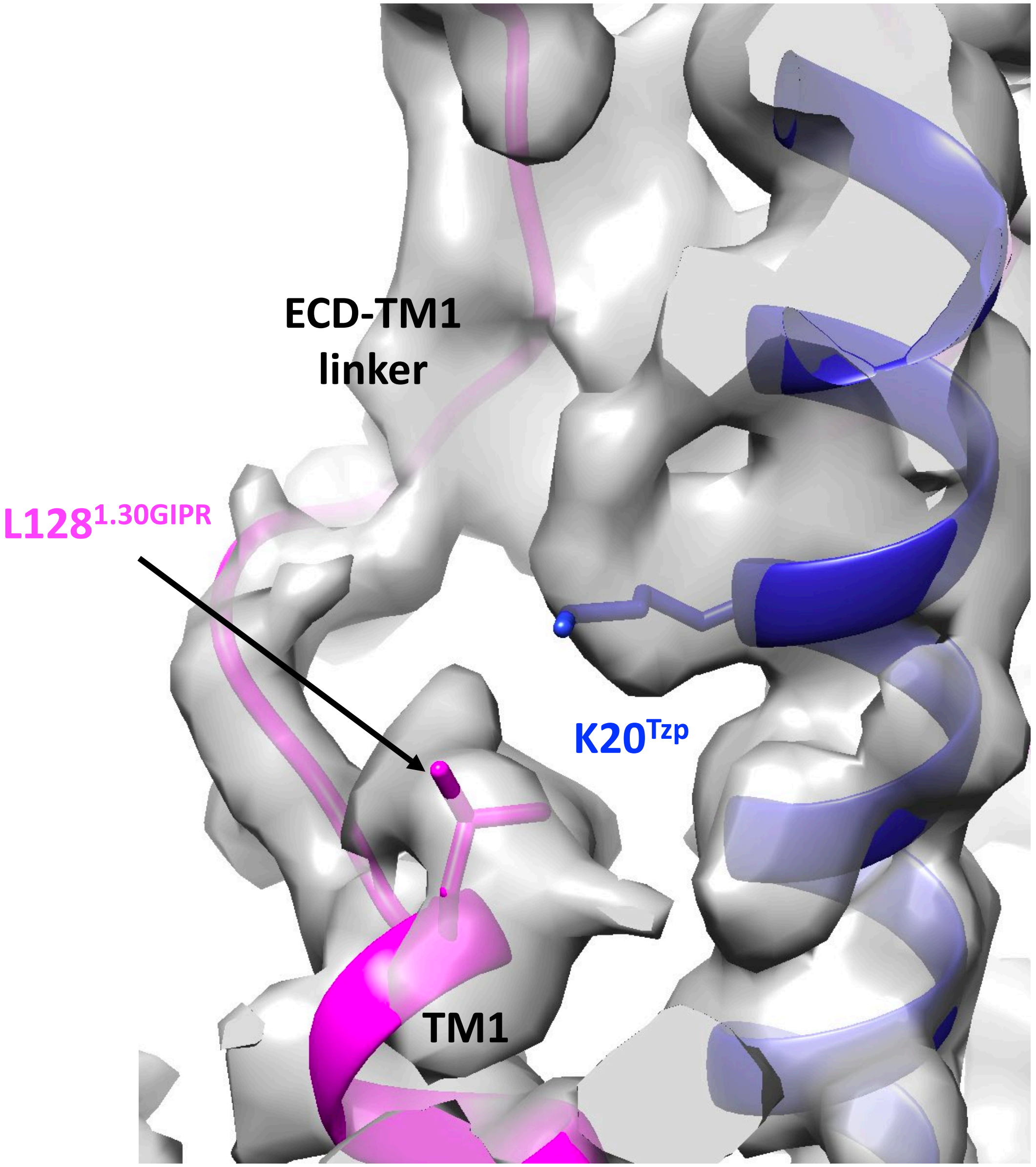


**Fig. S4. Lys20$^{Tzp}$ is located near the ECD-TM1 linker, specifically residue Leu128$^{1.30GIPR}$.** The fatty acid moiety attached to Lys20$^{Tzp}$ is not resolved in the density map. The map for ECD-TM1 linker is continuous, indicating reduced flexibility. The cryo-EM map is shown in silver color. Residues of Tzp are colored in blue, and residues of GIPR are colored in magenta.

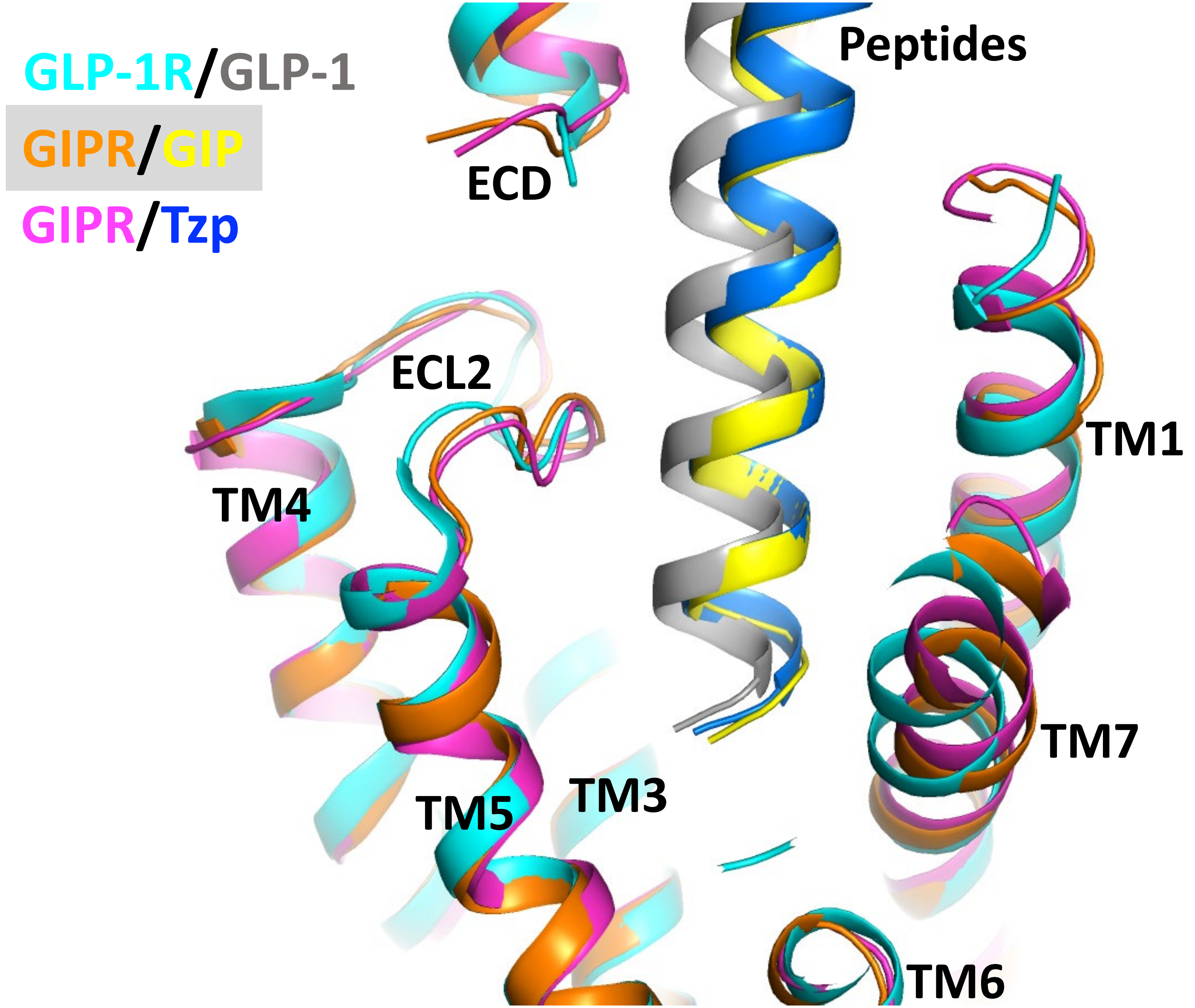


**Fig. S5. GIP and tirzepatide bind to the GIPR in a shifted position versus GLP-1 binding to the GLP-1R.** The structures of GIPR (orange) / GIP (yellow), GIPR (magenta) / Tzp (blue), and GLP-1R (cyan) / GLP-1 (gray) (PDB: 6X18), are aligned by the Cα atoms of the receptor 7TM. The peptide ligands of GIPR are located further away from ECL2 than GLP-1.

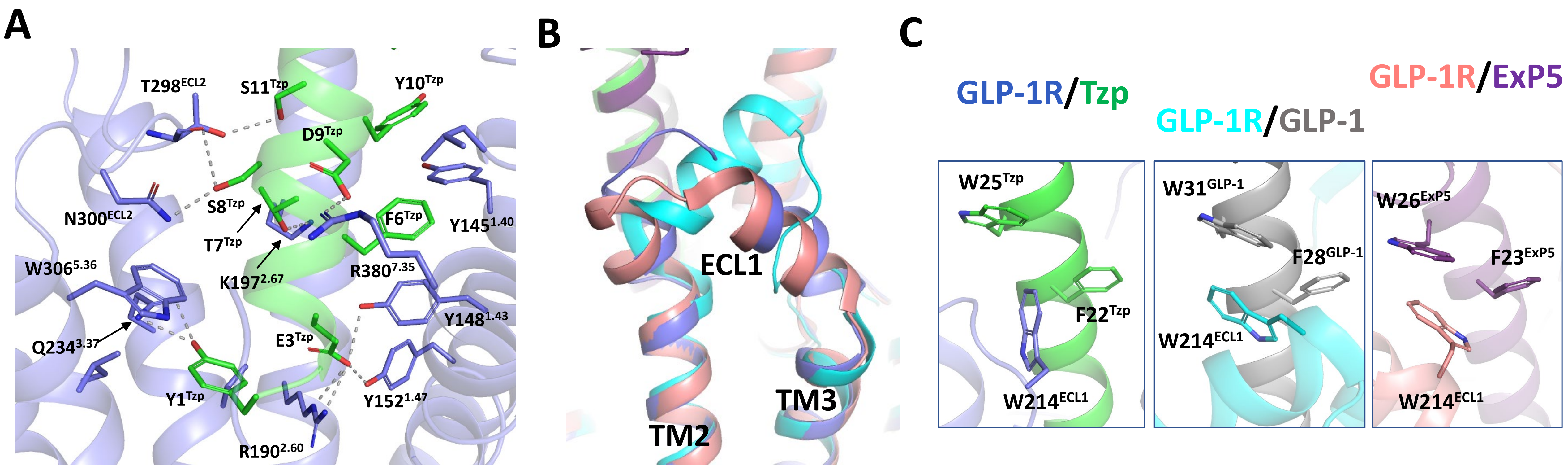


**Fig. S6. GLP-1R/tirzepatide interactions.** (A) The interaction of Tzp with the TM domain of the GIPR. Residues that are involved in interactions are shown in sticks and the residues that contribute most significant interactions are labeled. Hydrogen bonds were labeled in dashes. (B) The conformation of GLP-1R ECL1 when bound to Tzp (slate blue for GLP-1R, green for Tzp), GLP-1 (cyan for GLP-1R, gray for GLP-1), and ExP5 (salmon for GLP-1R, purple for ExP5). (C) The critical role of Trp214$^{ECL1}$ in interaction with the Phe-x-x-Trp motif of GLP-1R peptide ligands.

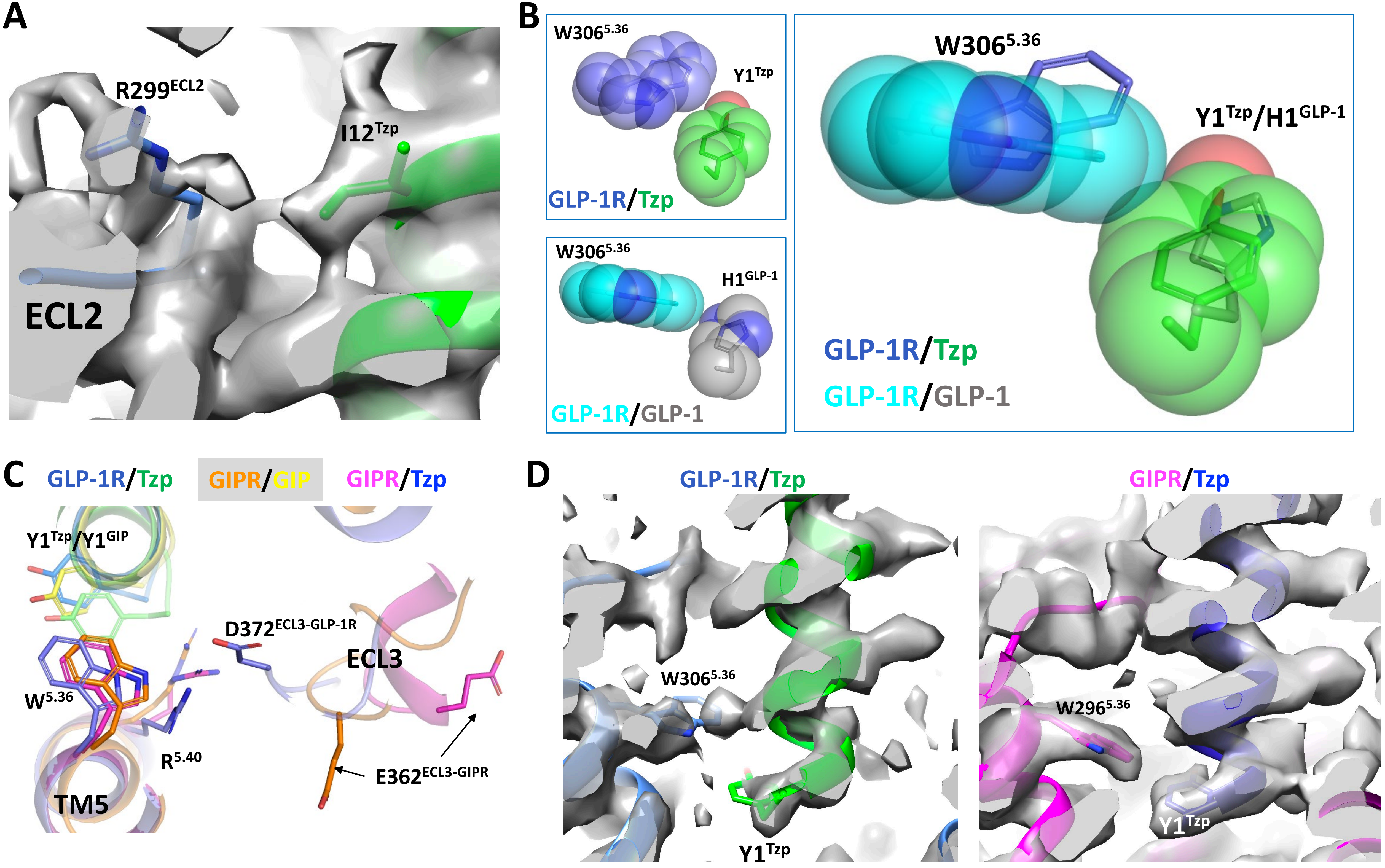


**Fig. S7. GLP-1R/tirzepatide structure features.** (A) The cryo-EM map of Arg299$^{ECL2}$ and its surroundings in the GLP-1R (slate blue) / Tzp (green) structure. There is no polar interaction between Arg299$^{ECL2}$ and Tzp. (B) The difference of rotamer of Trp306$^{5.36}$ in GLP-1R/Tzp and GLP-1R/GLP-1 structures, in response to the difference of residue 1 on the peptide. (C) The lack of interaction between TM5 and ECL3 in GLP-1R/Tzp, GIPR/GIP and GIPR/Tzp structures. (D) The cryo-EM map of Tyr1$^{Tzp}$ , Trp$^{5.36}$ and their surroundings in GLP-1R/Tzp and GIPR/Tzp structures. Tyr1$^{Tzp}$ and Trp$^{5.36}$ are less well defined in GLP-1R/Tzp structure comparing to GIPR/Tzp structure, while the maps for other surrounding residues are similar in quality, indicating less stable conformation.

A

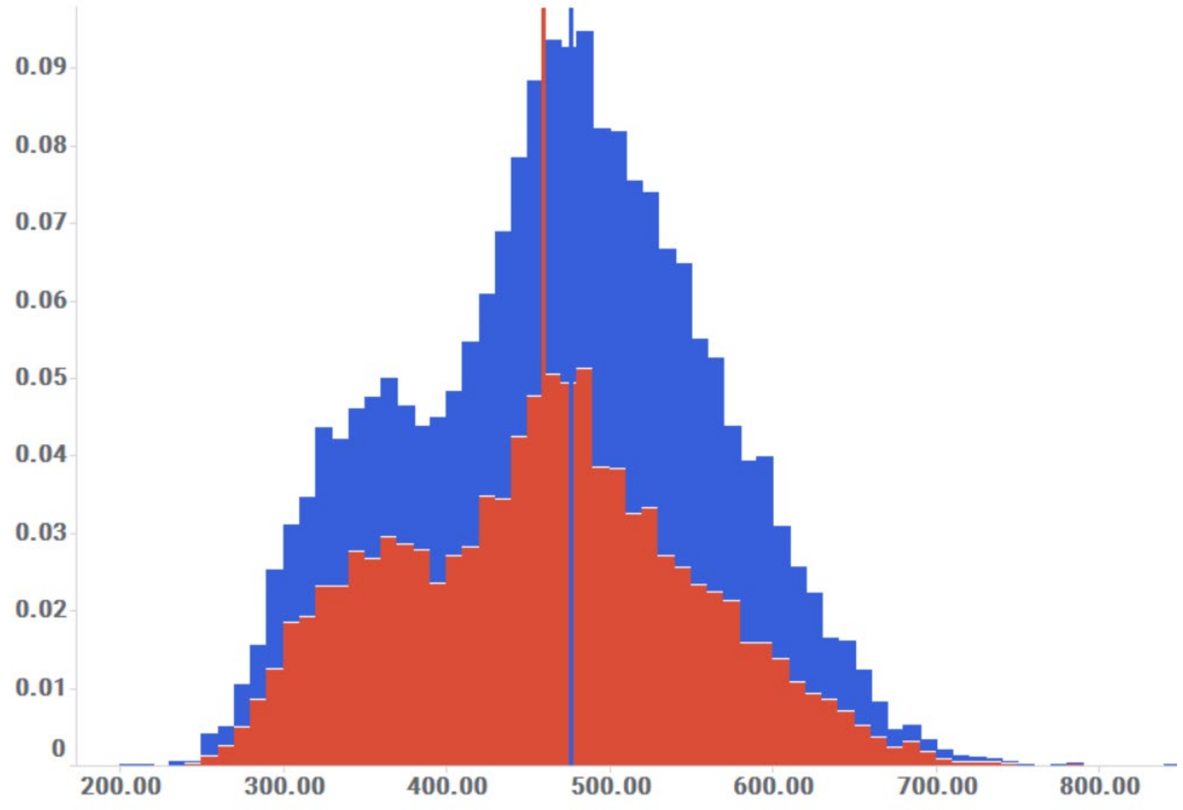

0.09
0.08
0.07
0.06
0.05
0.04
0.03
0.02
0.01
0
200.00
300.00
400.00
500.00
600.00
700.00
800.00


B

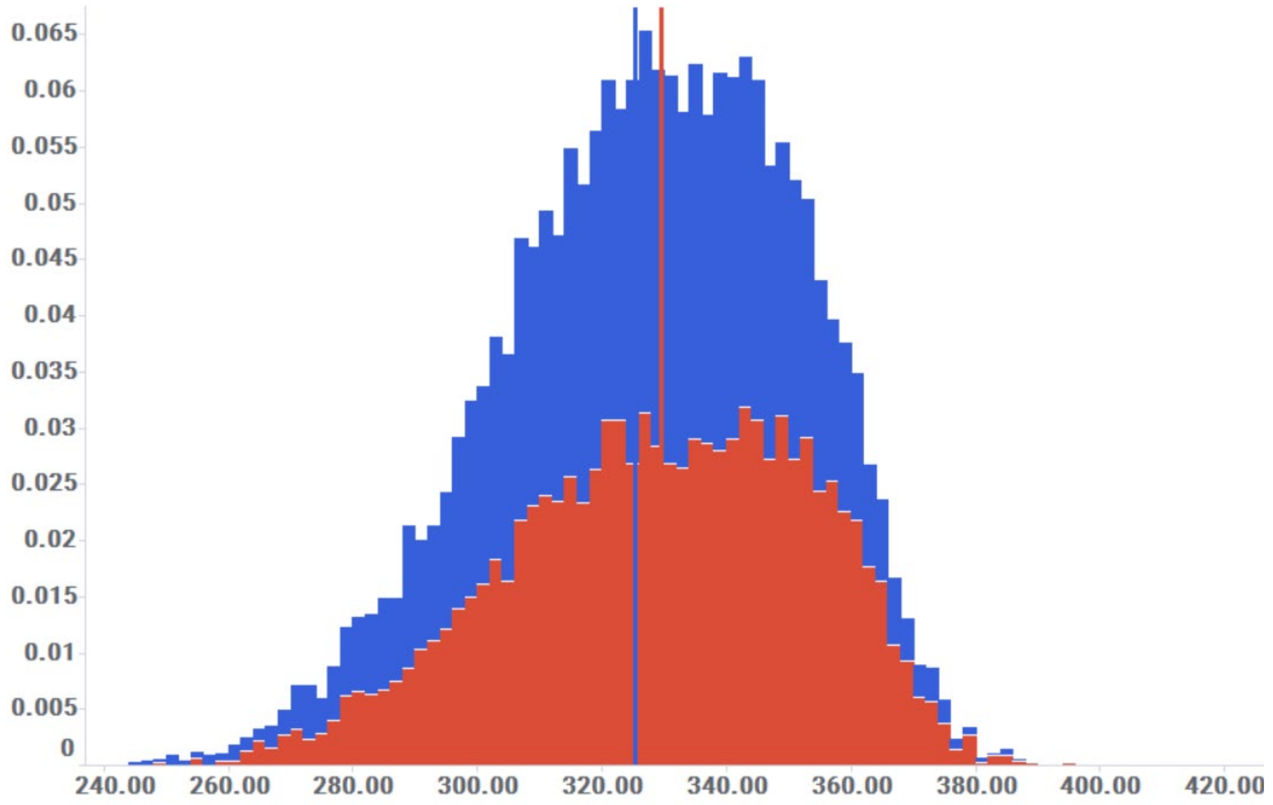

0.065
0.06
0.055
0.05
0.045
0.04
0.035
0.03
0.025
0.02
0.015
0.01
0.005
0
240.00
260.00
280.00
300.00
320.00
340.00
360.00
380.00
400.00
420.00

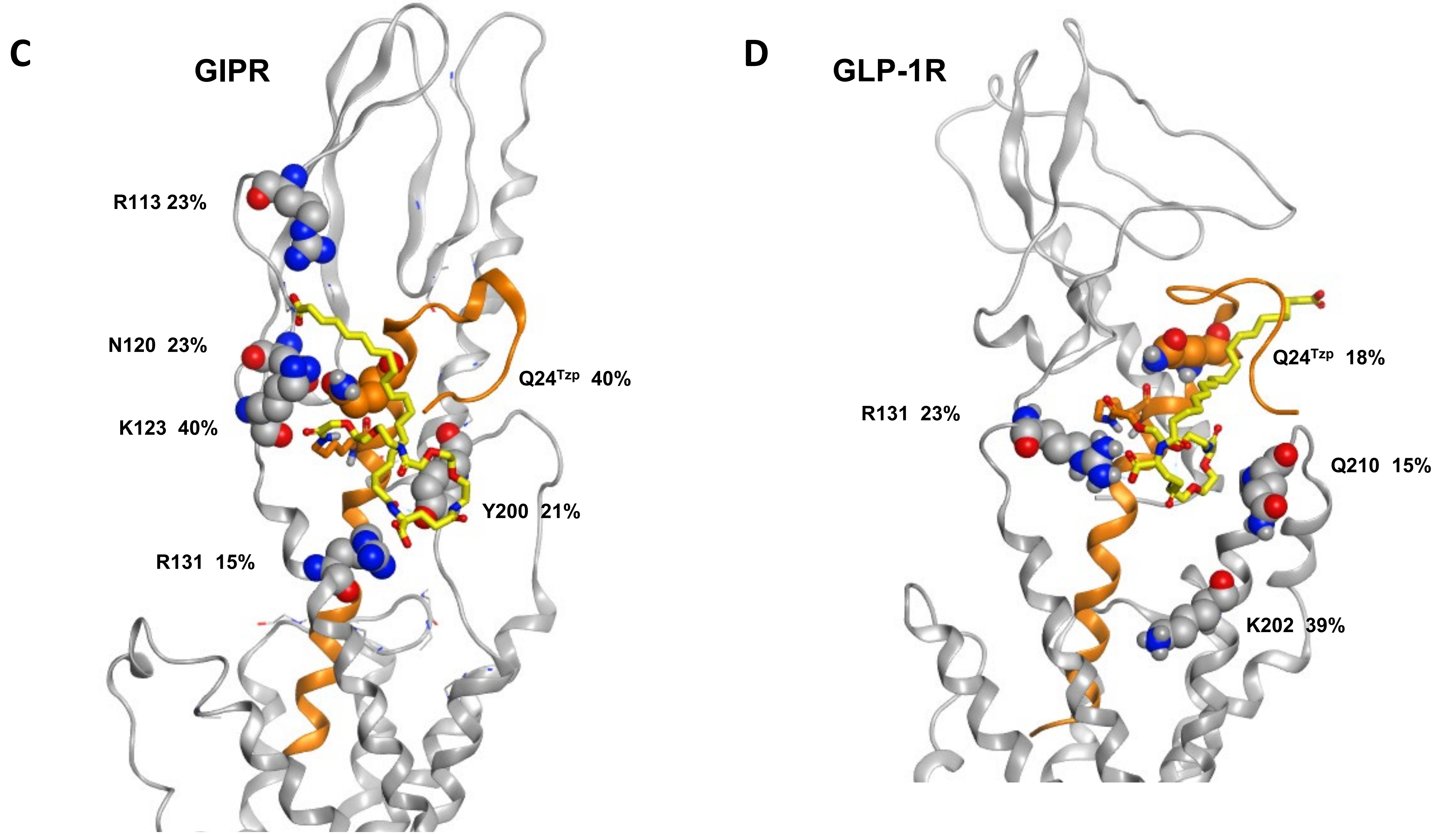


**Fig. S8. Assessment of solvent accessible and polar surface area.** (A) Solvent accessible surface area and (B) polar surface area distributions of the lipid chain over two 500 nsec MD runs in the GIPR (blue) and the GLP-1R (red) complexes, respectively. (C) GIPR and (D) GLP-1R: final frames from two 500 nsec simulations.

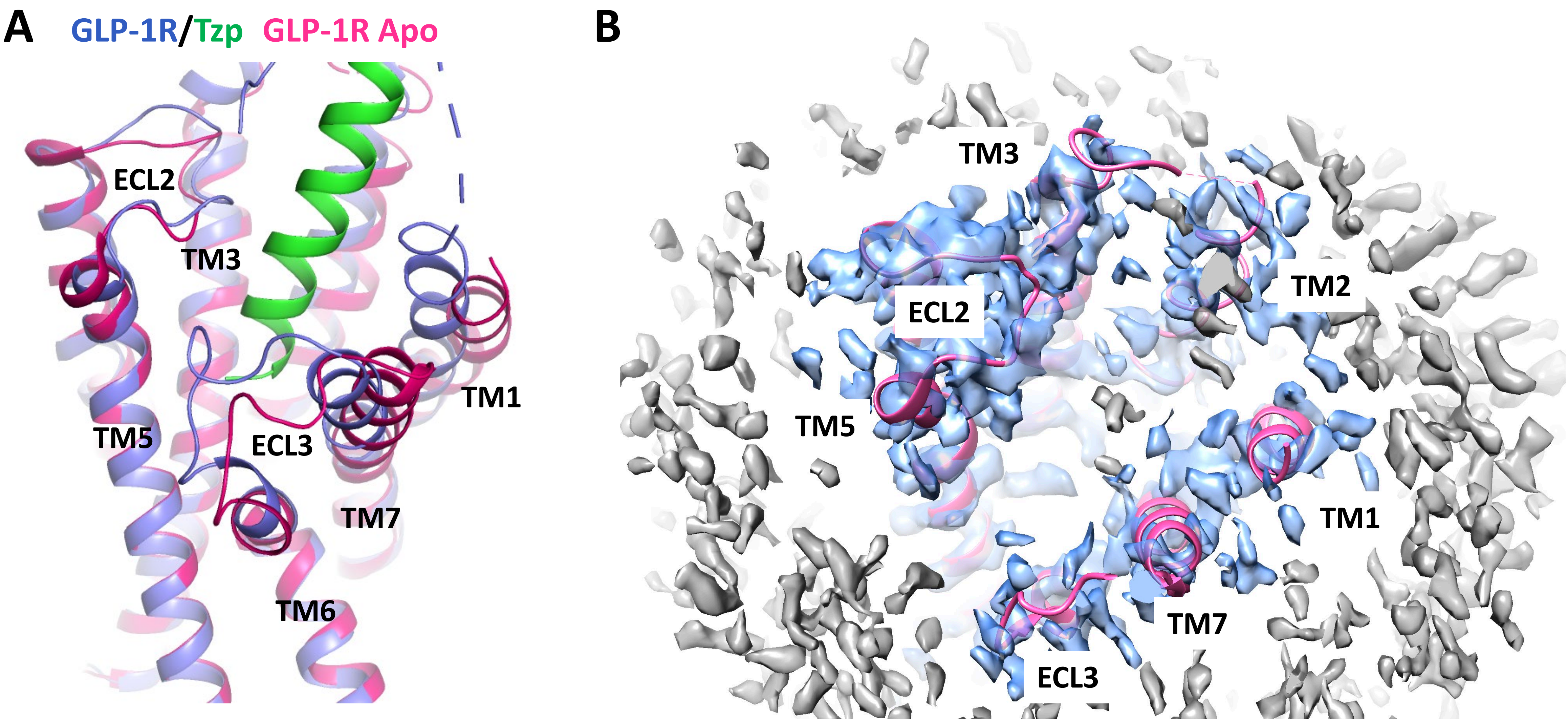


**Fig. S9. Structure of the GLP-1R apo-form (Apo) from the GLP-1R/tirzepatide sample.** (A) Overall structure alignment of GLP-1R Apo (pink) and GLP-1R (slate blue) / Tzp (green). Major TMs and ECLs are labeled. (B) The cryo-EM map revealed less well-defined extracellular portion of GLP-1R 7TM in the GLP-1R Apo structure, and no continuous density for Tzp. The density within 4 Å radius of the model are colored in blue, and other density are colored in silver.

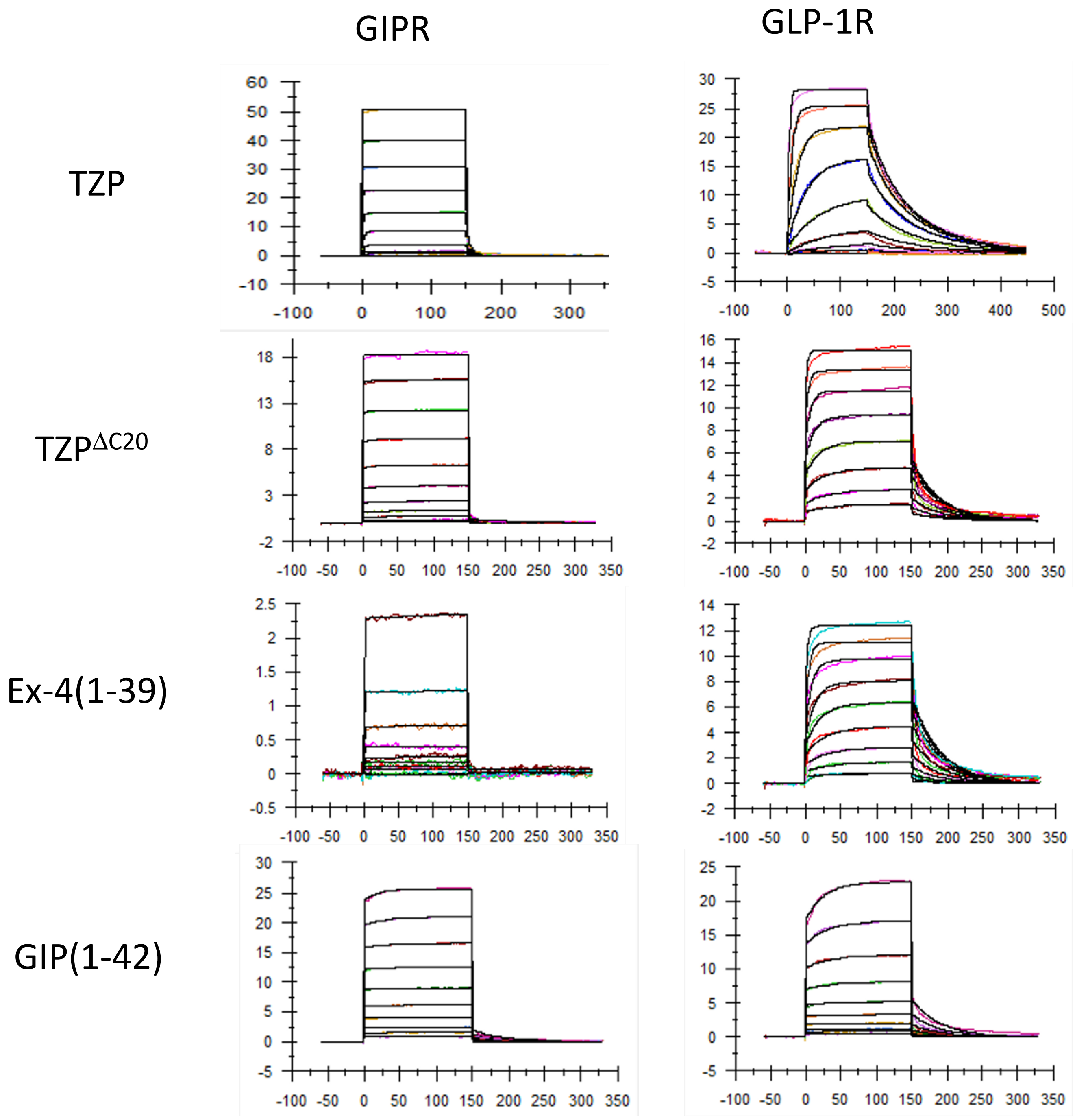

GIPR
GLP-1R
TZP
TZP^ΔC20
Ex-4(1-39)
GIP(1-42)

**Fig. S10. Receptor extracellular domain (ECD) binding affinity of peptides.** Purified GIPR (left column) and GLP-1R (right column) extracellular domains were immobilized to biosensor surfaces. Direct binding of peptides tirzepatide (TZP), de-lipidated tirzepatide $TZP^{(\Delta C20)}$, exendin-4(1-39) and GIP(1-42) was quantified by surface plasmon resonance. Peptides were tested at multiple concentrations with appropriate concentration ranges with a 2.5-fold dilution series. Maximum tested concentrations (GIPR, GLP-1R): TZP (49 mM, 410 nM), $TZP^{(\Delta C20)}$ (10 mM, 1.2 mM), exendin-4(1-39) (6 mM, 400 nM) and GIP(1-42) (31 mM, 125 mM). Data were fit to a 1:1 kinetic binding model (colored curve fits). Kinetic constants for GLP-1R ECD experiments were determined ($K_{on}$, $K_{off}$, $K_D$): TZP (9.42 x $10^5$ $M^{-1}s^{-1}$, 0.0182 $s^{-1}$, 1.93 x $10^{-8}$ M), $TZP^{(\Delta C20)}$ (3.60 x $10^5$ $M^{-1}s^{-1}$, 0.0254 $s^{-1}$, 7.06 x $10^{-8}$ M), exendin-4(1-39)(7.73 x $10^5$ $M^{-1}s^{-1}$, 0.0249 $s^{-1}$, 3.23 x $10^{-8}$ M), GIP(1-42) (No kinetic fit possible).  For interactions that reached equilibrium an equilibrium binding model was also fit (See Supplemental Table 2). Data shown are representative of n=3 independent determinations.

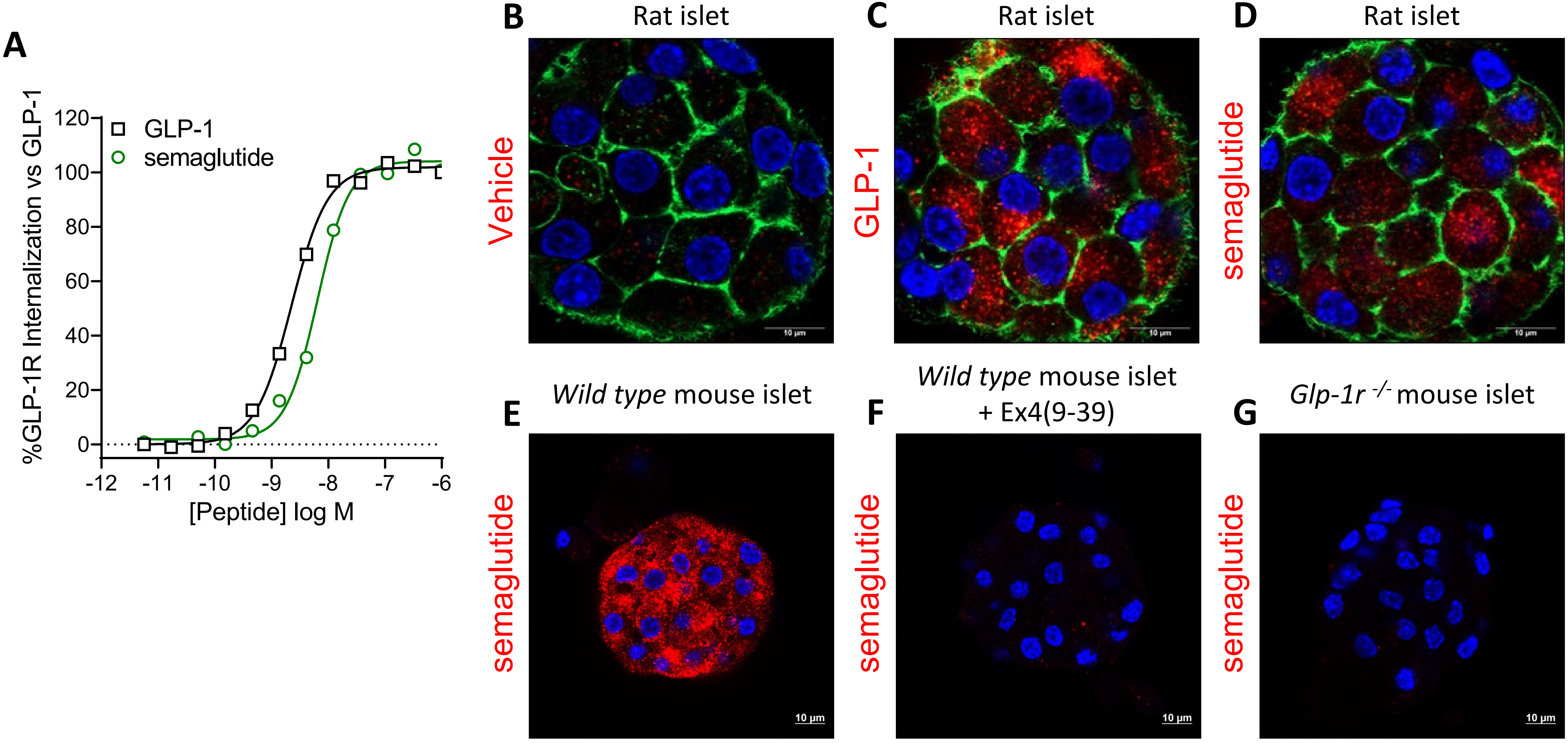

A
%GLP-1R Internalization vs GLP-1
120
100
80
60
40
20
0
-12
-11
-10
-9
-8
-7
-6
[Peptide] log M
GLP-1
semaglutide
B
Rat islet
Vehicle
C
Rat islet
GLP-1
D
Rat islet
semaglutide
E
*Wild type* mouse islet
semaglutide
10 µm
F
*Wild type* mouse islet + Ex4(9-39)
semaglutide
10 µm
G
*Glp-1r* -/- mouse islet
semaglutide
10 µm

**Fig. S11. Semaglutide-induced GLP-1R internalization in transgenic HEK293 cells and fluorescent semaglutide internalization in rat and mouse pancreatic islets.** (A) Ligand-induced internalization of the GLP-1R was assessed using changes in the cell surface presentation of SNAP-tagged receptor in HEK293 cells. Native GLP-1 and semaglutide are shown to be equally efficacious at inducing internalization of the GLP-1R. Data presented are representative of n≥3 independent experiments. (B-D) Representative confocal images of rat pancreatic islets. Fluorescence (red) was detected following incubation of islets with vehicle (B), 10 nM GLP-1$^{AF647}$ (C), or 10 nM semaglutide$^{AF647}$ (D) for 30 minutes. AlexaFluor488 Phalloidin staining (green) was used to detect cell membranes. (E-G) Representative confocal images of mouse pancreatic islets labelled with fluorescently-tagged semaglutide. Fluorescence (red) was detected following incubation of islets from wild type (E, F) or Glp-1r null (G) mice with 30 nM semaglutide$^{AF647}$ for 30 minutes. To block the GLP-1R, islets from wild type mice were pre-incubated with the GLP-1R antagonist exendin-4$_{(9\text{-}39)}$ (2 µM) (F) prior to treatment with the labelled semaglutide. Nuclei are stained in blue with Hoechst 33342. Bar, 10 µm.

## Table S1. Cryo-EM data collection, refinement and validation statistics

| | GIPR/GIP/$G_{siN18}$/Nb35/scFv16<br>EMD-24334; PDB: 7RA3 | GIPR/Tzp/$G_{siN18}$/Nb35/scFv16<br>EMD-24401; PDB: 7RBT | GLP-1R/Tzp/$G_{siN18}$/Nb35/scFv16<br>EMD-24453; PDB: 7RGP | GLP-1R/$G_{siN18}$/Nb35/scFv16<br>EMD-24445; PDB: 7RG9 |
|---|---|---|---|---|
| **Data collection and processing** | | | | |
| Voltage (kV) | 300 | 300 | 300 | 300 |
| Electron exposure (e–/Å$^2$) | 53.6 | 53.5 to 54.6 | 50.0 to 50.9 | 50.0 to 50.9 |
| Defocus range (μm) | -1.0 to -2.5 | -1.0 to -2.5 | -1.1 to -2.5 | -1.1 to -2.5 |
| Pixel size (Å) | 0.324 | 0.832 | 0.832 | 0.832 |
| Symmetry imposed | C1 | C1 | C1 | C1 |
| Initial particle images (no.) | 2,315,772 | 4,708,751 | 2,998,859 | 2,998,859 |
| Final particle images (no.) | 145,195 | 259,349 | 340,279 | 154,469 |
| Resolution (Å) (FSC 0.143 cutoff) | 3.2 | 3.1 | 2.9 | 3.2 |
| **Refinement** | | | | |
| Map sharpening *B* factor (Å$^2$) | -67.1 | -94.6 | -109.7 | -109.0 |
| *B* factors (Å$^2$) | 92.7 | 59.9 | 64.5 | 46.7 |
| Receptor | 125.0 | 78.8 | 96.2 | 70.2 |
| Peptide | 136.0 | 82.1 | 122.3 | N/A |
| R.m.s. deviations | | | | |
| Bond lengths (Å) | 0.005 | 0.006 | 0.006 | 0.011 |
| Bond angles (°) | 0.92 | 0.748 | 0.911 | 0.871 |
| MolProbity score | 1.83 | 2.28 | 2.24 | 2.20 |
| Clashscore | 6.90 | 18.0 | 17.5 | 15.5 |
| Poor rotamers (%) | 0.35 | 0.70 | 0.88 | 0.76 |
| Ramachandran plot | | | | |
| Favored (%) | 92.9 | 90.9 | 91.7 | 91.5 |
| Disallowed (%) | 0 | 0 | 0 | 0 |

## Table S2. GIPR and GLP-1R in vitro pharmacology

| Ligand | [125I]GIP Binding $K_i$ nM (SEM, n) | GIPR GTPγS Recruitment $EC_{50}$ nM (SEM, n) | GIPR GTPγS Recruitment Top (SEM) | GIPR Low Density cAMP $EC_{50}$ nM (SEM, n) | GIPR Low Density cAMP Top (SEM) | GIPR SPR $K_D$ nM (SEM, n) |
|---|---|---|---|---|---|---|
| **GIP** | 0.103 (0.011, 3) | 0.312 (0.136, 7) | 97.4 (3.6) | 0.355 (0.114, 6) | 99.4 (6.1) | 22,500 (800, 3) |
| **Exendin-4(1-39)** | ND | ND | ND | ND | ND | 6,700 (80, 3) |
| **GLP-1$^{H7Y}$** | >128 (ND, 3) | >1,000 (ND, 4) | ND | >4,000 (ND, 3) | 39.4(9.5) | ND |
| **Tzp $^{\Delta C20,Y1H}$** | 0.416 (0.043, 3) | 0.273 (0.209, 4) | 31.4 (6.6) | 0.682 (0.047, 4) | 80.9 (3.4) | ND |
| **Tzp$^{\Delta C20}$** | 0.0235 (0.0038, 3) | 0.0733 (0.0219, 4) | 91.0 (3.5) | 0.0163 (0.0032, 4) | 107 (4) | 1,690 (230, 3) |
| **Tzp** | 0.0870 (0.0098, 3) | 0.217 (0.048, 3) | 84.7 (2.1) | 0.319 (0.034, 4) | 105 (5) | 4,200 (220, 3) |

| Ligand | [125I]GLP-1 Binding $K_i$ nM (SEM, n) | GLP-1R GTPγS Recruitment $EC_{50}$ nM (SEM, n) | GLP-1R GTPγS Recruitment Top (SEM) | GLP-1R Low Density cAMP $EC_{50}$ nM (SEM, n) | GLP-1R Low Density cAMP Top (SEM) | GLP-1R SPR $K_D$ nM (SEM, n) |
|---|---|---|---|---|---|---|
| **GLP-1** | 0.594 (0.051, 3) | 0.240 (0.068, 8) | 103 (9) | 0.275 (0.024, 6) | 103 (5) | ND |
| **Exendin-4(1-39)** | ND | ND | ND | ND | ND | 35.6 (0.9, 3) |
| **GLP-1$^{H7Y}$** | 3.00 (0.28, 3) | 1.17 (0.33, 3) | 80.7 (2.2) | 9.32 (0.71, 4) | 104 (4) | ND |
| **Tzp $^{\Delta C20,Y1H}$** | 0.599 (0.083, 3) | 0.239 (0.092, 4) | 88.9 (3.9) | 0.163 (0.021, 4) | 111 (5) | ND |
| **Tzp$^{\Delta C20}$** | 2.67 (0.38, 3) | 0.626 (0.152, 4) | 74.8 (9.1) | 1.05 (0.06, 4) | 105 (2) | 111 (3, 3) |
| **Tzp** | 1.97 (0.47, 3) | 0.483 (0.270, 4) | 43.9 (1.7) | 7.07 (1.67, 4) | 99.5 (4.5) | 22.6 (1.6, 3) |

| Ligand | GLP-1R GRK2 Recruitment $EC_{50}$ nM (SEM, n) | GLP-1R GRK2 Recruitment Top (SEM) | GLP-1R β-Arr1 Recruitment $EC_{50}$ nM (SEM, n) | GLP-1R β-Arr1 Recruitment Top (SEM) | GLP-1R Internalization $EC_{50}$ nM (SEM, n) | GLP-1R Internalization Top (SEM) |
|---|---|---|---|---|---|---|
| **GLP-1** | 2.58 (0.35, 4) | 100 (NA) | 3.59 (0.21, 4) | 100 (NA) | 2.07 (0.60, 3) | 97.0 (4.4) |
| **GLP-1$^{H7Y}$** | 6.87 (0.75, 3) | 54.5 (2.1) | 9.60 (3.5, 4) | 35.3 (3.8) | 67.2 (17.6, 3) | 73.1 (3.4) |
| **Tzp $^{\Delta C20,Y1H}$** | 3.08 (1.65, 3) | 84.2 (1.5 ) | 3.19 (7.2, 4) | 74.6 (2.2) | 2.13 (0.74, 3) | 101 (6) |
| **Tzp$^{\Delta C20}$** | 1.92 (0.33, 3) | 39.4 (3.7) | 3.99 (0.64, 4) | 25.9 (2.6) | 5.47 (0.97, 3) | 90.6 (3.0) |
| **Tzp** | 5.92 (0.89, 3) | 24.7 (2.8 ) | 6.00 (1.63, 4) | 7.55 (0.93) | 36.5 (2.0, 3) | 41.6 (3.0) |

Top Panel: The affinity ($K_i$) of ligands for the GIPR was quantified by [$^{125}$I]GIP(1-42) competition binding using GIPR expressing cell membranes. Functional potency ($EC_{50}$) and efficacy (% Top) of ligands to induce $G\alpha_S$-GTPγS binding and cAMP accumulation in HEK293 cells expressing GIPR were determined. The equilibrium affinity ($K_D$) of ligands for the immobilized ectodomain of the GIPR were quantified by surface plasmon resonance. Middle Panel: The affinity ($K_i$) of ligands for the GLP-1R was quantified by [$^{125}$I]GLP-1(7-36) competition binding using GLP-1R expressing cell membranes. Functional potency ($EC_{50}$) and efficacy (% Top) of ligands to induce $G\alpha_S$-GTPγS binding and cAMP accumulation in HEK293 cells expressing GLP-1R were determined. The equilibrium affinity ($K_D$) of ligands for the immobilized ectodomain of the GLP-1R were quantified by surface plasmon resonance. Bottom Panel: Functional potency ($EC_{50}$) and efficacy (% Top) for ligands to induce GRK2, and β-arrestin1 recruitment to the GLP-1R were determined using the NanoBRET approach. The potency ($EC_{50}$) and efficacy (% Top) for ligands to induce GLP-1R Internalization were determined using diffusion-enhanced resonance energy transfer. Dose-response data were normalized to % stimulation using GIP(1-42) or GLP-1(7-36), or normalized by relative efficacy for NanoBRET assays. SPR data were analyzed using T200 evaluation software (Cytiva). All data are presented as geometric means with SEM and n values according to established practices. ND, not determined. NA, not applicable.
